# Validation of an improved insect bite hypersensitivity severity score for allergic equine insect bite hypersensitivity in horses

**DOI:** 10.1093/jvimsj/aalag132

**Published:** 2026-07-06

**Authors:** Juwela Lam, Anna Eckert, Tanya Rhiner, Nina Waldern, Elio Schwarz, Katharina Birkmann, Daniel Widmer, Macsmeila Dietrich, Thomas M Kündig, Antonia Fettelschoss-Gabriel

**Affiliations:** Evax AG, Guntershausen b. Aadorf, 8357, Switzerland; Department of Dermatology, University Hospital Zurich, Schlieren, 8952, Switzerland; Faculty of Medicine, University of Zurich, Zurich, 8032, Switzerland; Evax AG, Guntershausen b. Aadorf, 8357, Switzerland; Equine Department, Vetsuisse Faculty, University of Zurich, Zurich, 8057, Switzerland; Evax AG, Guntershausen b. Aadorf, 8357, Switzerland; Equine Department, Vetsuisse Faculty, University of Zurich, Zurich, 8057, Switzerland; Evax AG, Guntershausen b. Aadorf, 8357, Switzerland; Evax AG, Guntershausen b. Aadorf, 8357, Switzerland; Equine Department, Vetsuisse Faculty, University of Zurich, Zurich, 8057, Switzerland; Evax AG, Guntershausen b. Aadorf, 8357, Switzerland; Equine Clinic, Ludwig-Maximilians University Munich, Oberschleißheim, 85764, Germany; Evax AG, Guntershausen b. Aadorf, 8357, Switzerland; Department of Dermatology, University Hospital Zurich, Schlieren, 8952, Switzerland; Department of Dermatology, University Hospital Zurich, Schlieren, 8952, Switzerland; Faculty of Medicine, University of Zurich, Zurich, 8032, Switzerland; Evax AG, Guntershausen b. Aadorf, 8357, Switzerland; Department of Dermatology, University Hospital Zurich, Schlieren, 8952, Switzerland; Faculty of Medicine, University of Zurich, Zurich, 8032, Switzerland

**Keywords:** allergic equine insect bite hypersensitivity (IBH), Culicoides hypersensitivity, disease score, equine dermatology, observer agreement, therapeutic monitoring, validation

## Abstract

**Background:**

Insect bite hypersensitivity (IBH) is the most common allergic skin disease in horses, but existing clinical scoring systems lack extensive validation, limiting standardized disease monitoring and therapeutic evaluation.

**Hypothesis/Objectives:**

Develop and validate a standardized, sensitive, and reproducible clinical scoring system for IBH in horses.

**Animals:**

Forty-four privately-owned horses with clinically diagnosed IBH examined under field conditions in Switzerland.

**Methods:**

In this prospective field validation study, the equine IBH severity score (EqIS) integrates a lesion severity score and an area score using a multiplicative algorithm. Six trained evaluators assessed horses independently. Primary outcomes were intra- and interobserver reliability of the lesion severity score and EqIS assessed using intraclass correlation coefficients (ICC). Secondary analyses included agreement of the area score (Pearson correlation), correlations between owner-reported visual analog scale (VAS) scores and EqIS, and comparison between EqIS and a simplified EqIS developed for field use.

**Results:**

Intraobserver reliability was excellent (ICC = 0.995 for the lesion severity score and 0.989 for EqIS), and interobserver reliability was similarly high (ICC = 0.98 for both). The area score showed strong interobserver agreement (Pearson *r* = 0.86-0.94). Owner VAS scores correlated moderately with EqIS (ρ = 0.49-0.51) and strongly with each other (ρ = 0.88). The simplified EqIS demonstrated strong agreement with the validated version (*R*^2^ = 0.86).

**Conclusions and clinical importance:**

The EqIS is the first extensively validated IBH-specific scoring system and demonstrates excellent reproducibility across evaluators. It provides an objective tool for standardized disease assessment and therapeutic monitoring, whereas the simplified EqIS offers a practical alternative for routine field use.

## Introduction

Insect bite hypersensitivity (IBH) is the most frequent allergic skin disease of horses worldwide and represents a major welfare and management challenge, particularly in Icelandic horses imported to mainland Europe.^1^^,^^2^ The condition is triggered by *Culicoides* bites and is characterized by intense pruritus and regionally distributed skin lesions that typically flare seasonally.^3^ Because IBH is typically chronic and needs to be monitored over long periods, objective and reproducible quantification of clinical severity is essential for longitudinal assessment and evaluation of therapeutic interventions.^4–6^

Several clinical scoring systems have been used to standardize IBH assessment.^7–10^ Among these, the Fettelschoss-Miller scoring system has been widely and successfully applied in clinical trials and has demonstrated good ability to discriminate affected from non-affected horses, providing an important foundation for structured IBH lesion evaluation.^5^^,^^11^^,^^12^ However, most available IBH scores, including Fettelschoss-Miller, have not been formally validated across multiple evaluators, and systematic testing of intra- and interobserver reliability has not been reported.^4^^,^^11^ Beyond the lack of reproducibility testing, most available IBH scoring approaches rely on additive logic, summing lesion types or affected regions or both linearly. Although straightforward to apply, additive scores may underestimate clinically relevant severity when lesions are intense but localized, or conversely overestimate severity when many small, mild lesions are distributed across regions.^4^^,^^11^ Consequently, these factors limit comparability among studies and constrain the use of IBH scores as robust multi-observer endpoints for clinical trials and field studies.

In dermatology in humans, validated scoring systems that integrate lesion severity with affected surface area, using a multiplicative weighting, are widely used to improve objectivity and sensitivity to change.^13–15^ In veterinary dermatology, several validated indices exist for other diseases, but no extensively validated, IBH-specific instrument is available that combines severity and extent and has been tested across evaluators.^16–20^ We therefore developed a modular IBH scoring system centered on the equine IBH severity score (EqIS), which multiplies a lesion severity score with a regional area score using a multiplicative algorithm. To complement the veterinarian-assessed EqIS with welfare-relevant information, we additionally included owner-reported visual analog scales (VAS) for pruritus and overall IBH severity.^21^^,^^22^ The objectives of our field validation study were to evaluate inter- and intraobserver reliability of the lesion severity score and EqIS, assess agreement in scoring the extent of affected skin area using the area score, and examine associations between owner-reported VAS measures and EqIS. A simplified EqIS variant intended for routine field practice also is described.

## Materials and methods

### Horses and clinical study design

All study horses were client-owned. Studies were approved by the respective cantonal veterinary authorities (licenses 33558 and 34167), and owner informed consent was obtained. Horses were enrolled from a longitudinal monitoring cohort.

### Insect bite hypersensitivity scoring system

The EqIS combines a lesion severity score with an area score. Thirty-two predefined body areas were assessed for 7 lesion characteristics (broken hair, alopecia, blood or exudate, scales, crusts, lichenification, swelling) using a 0-4 ordinal severity scale. Alopecia, blood or exudate, and crusts were considered stand-alone lesions, whereas broken hair, scales, lichenification, and swelling required at least 2 concurrent findings for a body area to be classified as affected. The area score reflected the proportion of affected skin within each body area using a 6-point ordinal scale. For each body area, lesion severity scores were weighted using area score weighting factors (Table 1), and summed to yield the total EqIS. A simplified version of the EqIS was developed for field use using condensed lesion categories and simplified area weighting factors. Detailed scoring criteria, anatomical definitions, weighting factors, calculation methods, and scoring sheets are provided in Supplementary Methods and Figures S1-S4.

**Table 1:** Weighting factors used for calculation of the equine IBH severity score (EqIS).

**Area score category (form 35)**	**Weighting factor**
**Mild (1)**	0.5
**Mild–moderate (1.5)**	0.75
**Moderate (2)**	1.0
**Moderate–severe (2.5)**	1.25
**Severe (3)**	1.5

### Comparison instruments

The Fettelschoss-Miller scoring system was used as previously described^5^^,^^12^ and validated.^11^

### Validation procedures

All assessments were performed by trained veterinarians using photographic reference guides. For intraobserver reliability, 2 observers independently scored 12 IBH-affected horses (6 females, 6 males) twice on the same day (≥3 h apart) without intervening grooming or treatment. For interobserver reliability, 6 veterinarians independently scored 32 IBH-affected horses (10 females, 22 males) on the same day while blinded to each other’s assessments. Owner-assessed clinical outcomes for IBH severity and pruritus intensity were captured using 10 cm VAS. Detailed intraobserver, interobserver, and VAS procedures are provided in the Supplementary Methods.

### Statistical analysis

Analyses were conducted using GraphPad Prism 9.0 and R (irr package). Intraclass correlation coefficients (ICC) were used to assess intra- and interobserver reliability. Pearson correlation coefficients were used to assess agreement among observers and repeated measurements, whereas Spearman rank correlation coefficients were used to assess relationships between VAS scores and EqIS. Linear regression was used to evaluate agreement between EqIS and simplified EqIS and for comparison with the Fettelschoss-Miller score. Statistical significance was set at *P* < .05. The ICC agreement levels were interpreted according to benchmarks.^23^ Detailed statistical methods are provided in the Supplementary Methods.

## Results

### Intraobserver reliability

Intraobserver reliability was evaluated by repeated scoring of 12 IBH-affected horses by 2 observers on the same day.

**Figure 1:**
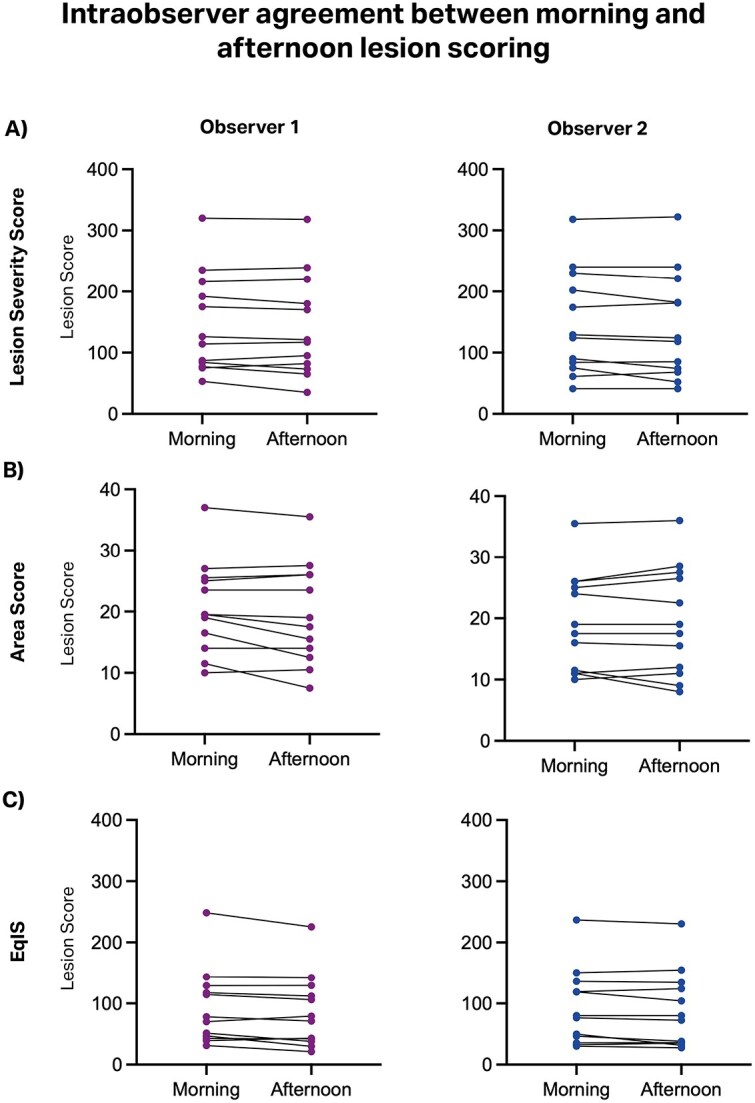
Individual IBH lesion scores per horse and time point (intraobserver assessment)*.* Paired dot plots showing the scores of 12 IBH-affected horses assessed by 2 observers at 2 time points on the same day (morning and afternoon) using (A) lesion severity score, (B) area score, and (C) equine IBH severity score (EqIS). Each dot represents one measurement; paired dots connected by lines represent repeated assessments of the same horse.

**Figure 2:**
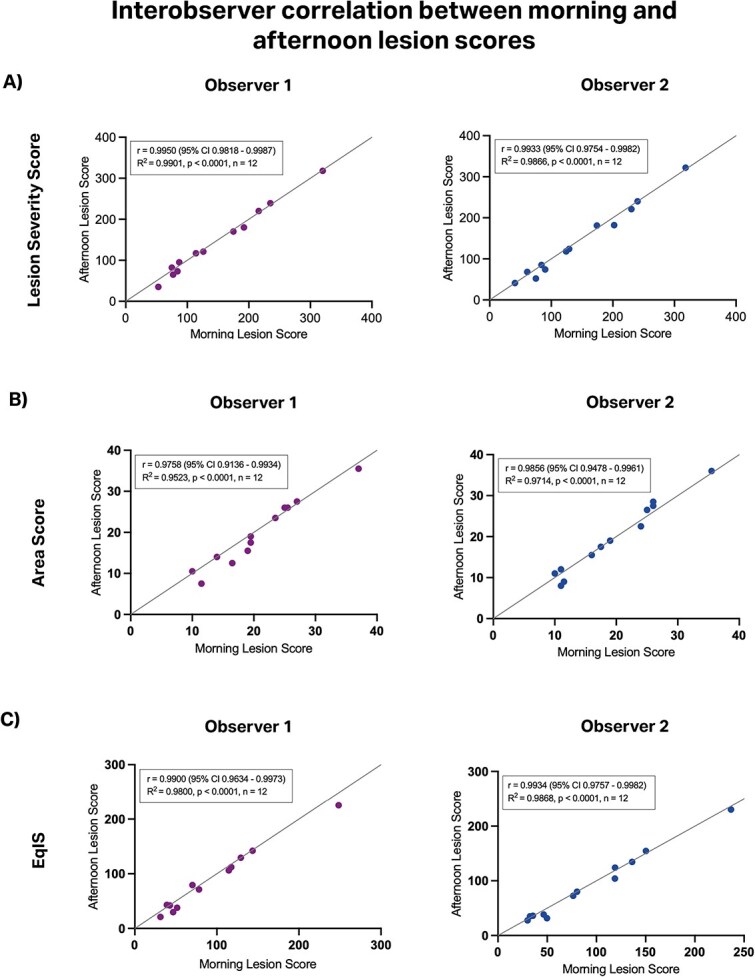
Intraobserver reliability of the IBH scoring system. Pearson correlation plots comparing morning and afternoon scores for each observer using (A) lesion severity score, (B) area score, and (C) equine IBH severity score (EqIS). Each dot represents one IBH-affected horse (*n* = 12). The solid black line represents the line of identity (*y* = *x*). Panels display Pearson’s correlation coefficient (*r*), coefficient of determination (*R*^2^), 95% CI, and *P*-values.

Paired measurements are shown in Figure 1. Agreement was excellent for all variables, with Pearson correlation coefficients exceeding 0.97 for all scores and ICC exceeding 0.98 for lesion severity score and EqIS (Table 2). All correlations were highly significant (*P* < .0001). Agreement among repeated measurements, as assessed by Pearson correlation coefficients, is illustrated in Figure 2.

**Table 2:** Intraobserver reliability of the lesion severity score, area score, and EqIS.

**Score**	**Observer**	**Pearson *r* (95% CI)**	**ICC (95% CI)**
Lesion severity score	1	0.9950 (0.9818-0.9987)	0.9946 (0.9814-0.9984)
	2	0.9933 (0.9754-0.9982)	0.9931 (0.9761-0.9980)
Area score	1	0.9758 (0.9168-0.9934)	–
	2	0.9856 (0.9478-0.9961)	–
EqIS	1	0.9900 (0.9634-0.9973)	0.9890 (0.9623-0.9968)
	2	0.9934 (0.9757-0.9982)	0.9933 (0.9769-0.9981)

All correlations were statistically significant (*P* < .0001).

### Interobserver reliability

**Figure 3:**
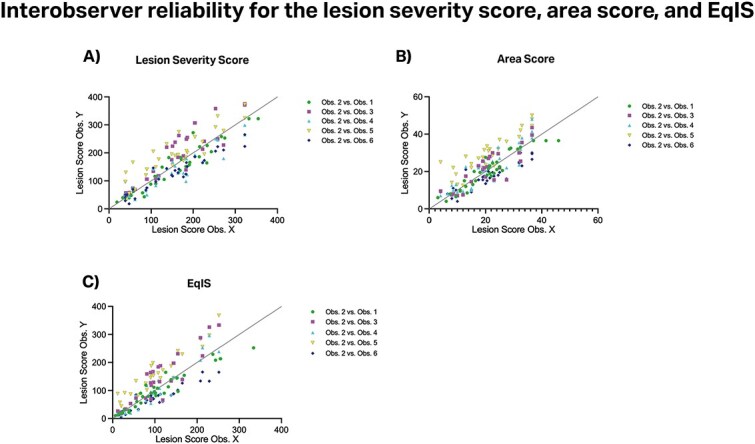
Interobserver reliability of the IBH scoring system. For visual clarity, Pearson correlation scatter plots illustrate only the comparisons between observer 2 and the remaining observers (observers 1 and 3-6) for (A) lesion severity score, (B) area score, and (C) equine IBH severity score (EqIS). Each point represents one IBH-affected horse (*n* = 28-32 depending on observer pair). The solid black line represents the line of identity (*y* = *x*). Complete Pearson correlation statistics (*r*, 95% CI, *R*^2^, *P*-values, and sample sizes) for all observer pairs are provided in the Tables S1-S3.

Interobserver reliability was assessed by 6 trained veterinarians (observers 1-6) who independently evaluated 32 IBH-affected horses. Correlation analyses were performed for all possible observer pairs. For visual clarity, Figure 3 presents comparisons between observer 2 and the remaining observers (observers 1 and 3-6), and complete correlation results are provided Tables S1-S3.

Correlation coefficients ranged from 0.86 to 0.96 across all variables. All correlations were significant (*P* < .0001). ICC indicated excellent agreement for lesion severity score and EqIS (Table 3).

**Table 3:** Interobserver reliability of the lesion severity score, area score, and EqIS.

**Score**	**Pearson *r***	**ICC (95% CI)**
**Lesion severity score**	0.88-0.96	0.98 (0.97-0.99)
**Area score**	0.86-0.94	–
**EqIS**	0.87-0.96	0.98 (0.96-0.99)

All correlations were statistically significant (*P* < .0001).

### Correlation of owner-reported VAS scores with EqIS

**Figure 4:**
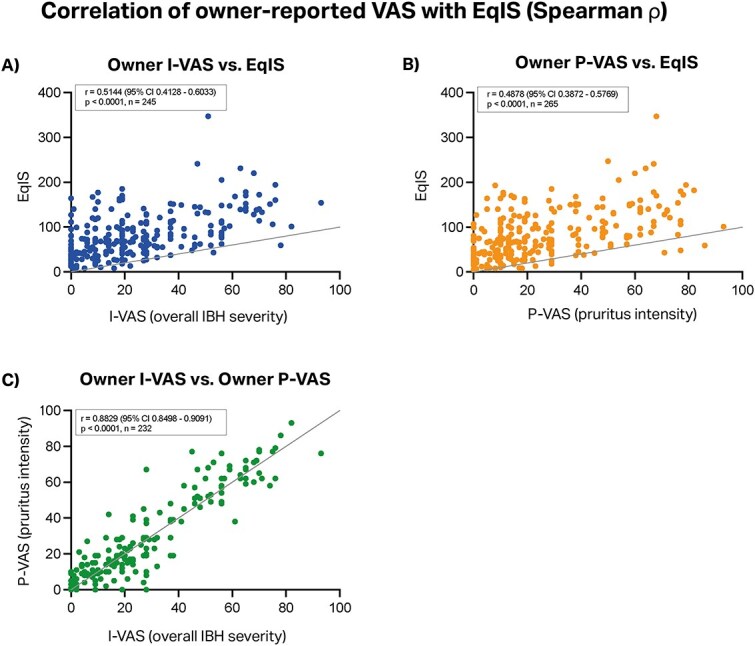
Correlation of owner-reported VAS scores with EqIS. (A) Correlation between owner-assessed VAS for overall IBH severity (I-VAS) and veterinarian-derived EqIS. (B) Correlation between owner-assessed VAS for pruritus intensity (P-VAS) and EqIS. (C) Correlation between the 2 owner-reported VAS measures. Spearman’s rank correlation coefficients (ρ), 95% CI, and *P*-values are shown. The solid line represents the line of identity (*y* = *x*).

Spearman rank correlation was used to evaluate associations between owner-reported VAS scores and veterinarian-assessed EqIS (Figure 4).

The VAS for overall IBH severity showed a moderate positive correlation with EqIS (ρ = 0.5144; 95% CI, 0.4128-0.6033; *P* < .0001).

The VAS for pruritus intensity correlated moderately with EqIS (ρ = 0.4878; 95% CI, 0.3872-0.5769; *P* < .0001).

The 2 owner-reported VAS measures were strongly correlated (ρ = 0.8829; 95% CI, 0.8498-0.9091; *P* < .0001).

### Comparison of validated and simplified EqIS and Fettelschoss-Miller score

The validated EqIS, the simplified EqIS, and the previously established Fettelschoss-Miller score were applied in parallel to the 2025 IBH clinical dataset. Regression analysis identified a strong relationship between the validated and simplified EqIS (*R*^2^ = 0.86; Figure S5A).

The relationship between the validated EqIS and the Fettelschoss-Miller score was lower (*R*^2^ = 0.73; Figure S5B).

A direct comparison of all 3 scoring systems is shown in Figure S5C.

## Discussion

Validated clinical scoring systems are well established in equine medicine for a range of conditions, including lameness, gastric ulcers, and neurologic deficits.^24–29^ In contrast, standardized and validated dermatologic assessment tools for horses remain limited. Although the equine urticaria activity score (EqUAS) provides a structured method for quantifying urticaria,^16^ it does not encompass the complex and multifaceted clinical features of IBH. Existing IBH scoring approaches have contributed valuable frameworks for clinical research, but most rely on simple additive scoring models and have not undergone formal evaluation of intra- and interobserver reliability. Limited validation may decrease reproducibility and sensitivity, particularly in cases with marked regional variation in lesion distribution.^4^^,^^11^^,^^30^

To address these limitations, we developed and validated a modular IBH scoring system, specifically designed to capture the full clinical spectrum of IBH in horses. The EqIS integrated region-based lesion assessment with quantification of affected surface area using a multiplicative approach. Multidimensional scoring strategies combining lesion severity and affected area are well established in dermatology in humans (eg, eczema area and severity index, EASI; psoriasis area and severity index, PASI) and are considered to improve standardization and sensitivity to change and standardization of disease assessment.^14^^,^^15^ To our knowledge, ours is among the first IBH-specific scoring systems in equine dermatology to integrate these dimensions while undergoing formal evaluation of observer reliability and clinical applicability.

### Reliability and reproducibility

Robust reproducibility is essential when clinical scoring systems are used as outcome measures in clinical trials, longitudinal monitoring, or therapeutic evaluation.^29^^,^^31^^,^^32^ In our study, lesion severity scoring and the composite EqIS score had outstanding reliability, with Pearson correlation coefficients and ICCs consistently exceeding 0.90 for both intra- and interobserver assessments. According to established guidelines, ICC values above 0.90 are considered excellent.^23^ Agreement for the area score was similarly high, with intraobserver Pearson correlations of approximately 0.98 and interobserver values between 0.88 and 0.94. As observed in other clinical scoring systems, intraobserver reliability was slightly higher than interobserver reliability, although the differences were small.^24^^,^^29^^,^^33^^,^^34^ This pattern likely reflects the fact that individual observers tend to apply and interpret the grading system more consistently over time, whereas slight variations in interpretation can occur among different observers.^29^^,^^35^ The high level of agreement observed in our study supports the use of standardized training, defined scoring criteria, and a detailed photographic reference glossary to promote consistent lesion interpretation.^4^^,^^29^

An important implication of these findings is that high interobserver agreement was achieved despite differences in prior experience among observers. Notably, 2 of the 6 veterinarians had limited previous exposure to IBH lesion scoring, yet reliability remained high after standardized training. This result suggests that the scoring system can be applied consistently after appropriate training, which is relevant for broader use in field settings.

Taken together, these results support the robustness and reproducibility of the IBH scoring system under field conditions. The reliability observed for all scoring forms is consistent with requirements for use as outcome measures in clinical studies.^23^^,^^29^^,^^31^^,^^32^

### Methodological context and comparison with established scoring systems

When placed in the broader context of validated dermatologic scoring systems in both veterinary and human medicine, the EqIS demonstrated high observer reliability and methodological features consistent with established instruments. Similar to veterinary dermatology scoring systems such as CADESI and EqUAS,^16^^,^^18–20^ EqIS records lesion morphology and anatomical distribution. In addition, EqIS incorporates affected surface area using multiplicative weighting, paralleling scoring systems used in humans such as EASI and PASI, which combine lesion severity with surface area involvement.^14^^,^^15^

**Figure 5:**
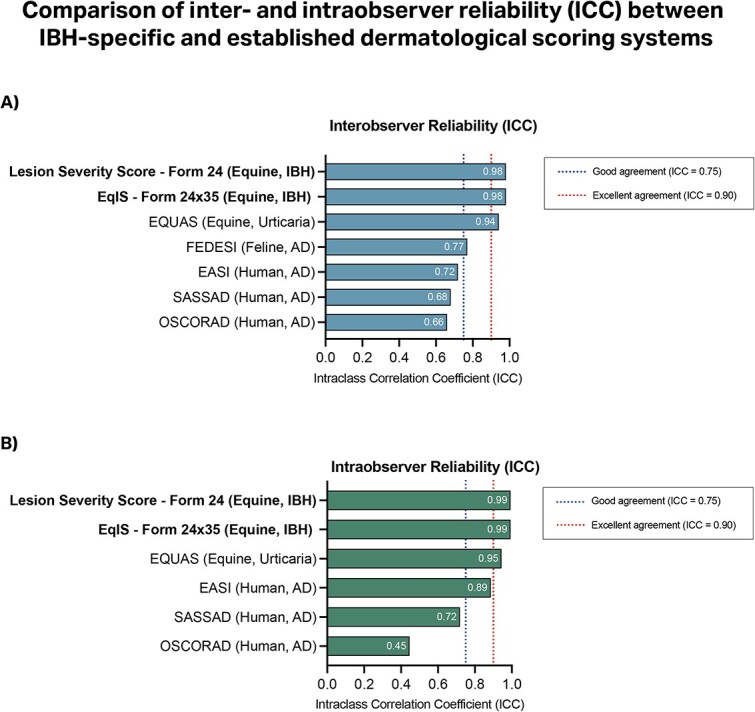
Comparison of intra- and interobserver reliability between established dermatological scoring systems and validated IBH-specific scores. (A) Interobserver reliability (intraclass correlation coefficient, ICC) for scoring systems used in human (oSCORAD, SASSAD, EASI), feline (FEDESI), and equine (EqUAS) dermatology compared with IBH-specific scoring tools (EqIS) and the lesion severity score. (B) Intraobserver reliability for the same scoring systems. Bars are sorted in descending order by ICC value within each panel. Vertical dotted lines indicate commonly accepted thresholds for “good” agreement (ICC = 0.75) and “excellent” agreement (ICC = 0.90). Published ICC values were obtained from the literature,^13–16^^,^^36^^,^^37^ whereas IBH-specific scores were calculated in our study.

To contextualize reliability, intra- and interobserver ICCs obtained in our study were compared with published ICCs for established dermatologic scoring systems (Figure 5). In our study, both intra- and interobserver ICCs for the lesion severity score and EqIS were high, indicating consistent scoring within and between observers. Sources for the comparative ICC values are provided in the Figure 5 legend.

Although cross-species and cross-disease comparisons should be interpreted cautiously. Figure 5 places the reliability of the IBH scoring system within the range reported for widely used dermatologic scoring instruments. Because no extensively validated IBH-specific scoring system has been available previously,^4^^,^^11^ these findings support the use of EqIS as a standardized outcome measure in clinical studies.

### Owner-reported VAS and EqIS

Owner-reported VAS scores provided complementary information to the EqIS. The strong correlation between owner-assessed pruritus intensity and overall disease severity reflects that owners primarily judge disease severity based on the intensity of pruritus, which is the most immediately observable and distressing manifestation of IBH. This observation is consistent with the central role of pruritus in behavior and welfare and aligns with findings in other species in which pruritus is a main driver of perceived disease severity.^17^^,^^22^

Correlations between owner-rated IBH severity and EqIS were moderate (*r* ≈ 0.49-0.51), indicating that owners and clinicians capture overlapping but not identical aspects of disease expression. Similar patterns are reported in veterinary and human medical dermatology, where owner- or patient-reported outcomes reflect subjective disease burden, whereas clinician-based scores assess objective lesion characteristics.^17–19^^,^^36^^,^^38^

Several factors may contribute to this moderate agreement. First, the EqIS is a highly detailed and objective scoring system that captures subtle morphological changes, including small but clinically relevant lesions, which may not be perceived as equally important by owners. As a result, EqIS values may exceed owner-reported VAS values, particularly in cases with very small but morphologically complex lesions. Comparable discrepancies have been described for highly granular dermatologic scoring systems.^37–40^

Second, differences in the temporal framework of assessment may have further contributed to moderate correlation. Owner-reported VAS scores were obtained repeatedly over time and therefore reflect a more integrated perception of disease severity across the observation period, rather than a single time point. In contrast, the EqIS represents a clinical assessment at a defined time point. Given that IBH severity can fluctuate considerably because of environmental exposure, weather conditions, insect activity, and self-trauma, this mismatch in temporal resolution may have attenuated the observed correlation between owner-reported and clinician-based measures.

Taken together, these findings emphasize the complementary value of both approaches and help explain the moderate correlation observed between them. Owner-reported VAS scores reflect a more integrated perception of disease severity over time and emphasize clinically apparent features such as pruritus, whereas the EqIS provides an objective and reproducible assessment of detailed lesion characteristics at a defined time point. Incorporating both perspectives may improve clinical monitoring and overall assessment of IBH.

### Strengths and limitations

The EqIS represents the first extensively validated scoring system for IBH, demonstrating excellent intra- and interobserver reliability across scorers with different levels of experience. Its modular, region-based, and multiplicative structure allows objective quantification of both lesion severity and distribution, supporting reproducibility and sensitivity for clinically meaningful changes. These features make the EqIS suitable for standardized application in clinical studies and controlled trials, where objectivity and reproducibility are of primary importance.^31^

A potential limitation of the intraobserver reliability assessment is that repeated scoring was performed on the same day after a relatively short interval. Although this approach minimized true biological variation in lesion appearance, it may have introduced recall bias and therefore could have led to a slight overestimation of intraobserver agreement. This design represents a methodological trade-off, because longer intervals between assessments would likely decrease recall bias but increase the risk of true changes in lesion appearance. In IBH, this possibility is particularly relevant because lesion severity can change rapidly because of self-trauma associated with pruritus, such as rubbing or scratching, as well as ongoing environmental exposure. As a result, longer intervals may introduce additional variability unrelated to observer consistency.

In addition to these methodological considerations, certain limitations are inherent to the design of the scoring system itself. The EqIS is a highly detailed scoring system, incorporating numerous small regional and morphological categories to capture the full spectrum of IBH lesion patterns. This high level of granularity enhances precision and consistency among scorers but also may represent a limitation in certain situations. Very small but morphologically complex lesions can contribute disproportionately to total scores, potentially resulting in higher overall values.^11^ Although this design enhances precision, it may not always capture the overall clinical picture of IBH, particularly from an experienced clinician’s perspective, and may limit detection of early changes during therapeutic monitoring. Similar limitations have been described for highly granular dermatologic scoring systems in human medicine.^13^^,^^41^^,^^42^

To address this aspect, a simplified version of the EqIS was developed as a practical adaptation of the validated system. This version keeps the same modular and multiplicative structure while allowing experienced scorers slightly more flexibility when assessing small or localized lesions. Such flexibility may improve alignment between numerical scores and overall clinical impression without altering the underlying scoring principle.

### Refinement and practical evaluation of the simplified EqIS

The simplified EqIS was developed to decrease potential overemphasis on small but morphologically complex lesions while preserving the analytical logic of the validated system. Both the validated and simplified EqIS were applied in parallel to the 2025 IBH clinical dataset to evaluate agreement and practical applicability.

Regression analyses demonstrated a strong relationship between the simplified and validated EqIS, indicating preservation of the measurement principles of the original system. The relationship between the validated EqIS and the Fettelschoss-Miller score was lower but remained substantial. Direct comparison showed closer alignment between the validated and simplified EqIS, likely reflecting their shared modular and multiplicative logic, whereas the Fettelschoss-Miller system is based on an additive design.

These findings indicate that the simplified EqIS maintains analytical agreement with the validated version while introducing a small degree of flexibility in scoring. Although the validated EqIS provides a highly objective and standardized assessment, the simplified version allows experienced scorers to better account for the overall clinical picture, particularly in cases with small but highly reactive lesions.^11^^,^^13^ In practice, the simplified EqIS can be applied more rapidly and may facilitate scoring under field conditions and in longitudinal studies while maintaining comparability with the validated system.

Because the simplified approach introduces limited scorer flexibility, consistent application requires familiarity with IBH lesion patterns and adherence to standardized scoring principles.^41^^,^^42^ When used by trained scorers, the simplified EqIS may support efficient data while preserving clinical relevance. Further evaluation is needed to assess its reproducibility across different observer groups and geographic settings. Geographic variation in lesion distribution may influence how scorers apply and interpret the system in practice.

### Geographic variation and contextual interpretation

Although the simplified EqIS decreases the potential overemphasis of small but highly affected regions, score interpretation remains context-dependent because lesion patterns vary with regional *Culicoides* ecology.^10^^,^^43^

In Europe, where *Culicoides obsoletus* and *Culicoides scoticus* predominate, lesions typically cluster along the dorsal midline, typically affecting the mane and tail base, with frequent additional involvement of the ventral midline (abdomen, udder or preputium), head, and ears.^2^^,^^10^^,^^43–47^ Outside Europe, *Culicoides sonorensis* in North America is linked mainly to the mane, withers, and tail base, whereas ventral lesions are uncommon.^47–49^ A comparable pattern is seen in Australia, where *Culicoides brevitarsis* predominates. Lesions mainly involve the mane, back, and tail base, very rarely extending to the head or ears, whereas ventral lesions are uncommon.^4^

These regional differences are clinically relevant because small but intensely affected areas (eg, ears or ventral midline) may reach high severity scores despite limited surface area, whereas larger ventral abdominal lesions often may cover larger areas but seldom achieve maximal severity. As a result, similar total scores may reflect different clinical pictures depending on regional lesion distribution.

Accordingly, interpretation of IBH scoring results should consider regional lesion patterns. Familiarity with locally prevalent lesion distributions may improve scoring consistency and interpretation across different geographic settings. The simplified EqIS may facilitate this context-sensitive assessment by allowing scorers to account for the overall clinical distribution of lesions.

In conclusion, the EqIS provides an IBH-specific, standardized instrument with excellent intra- and interobserver reliability under field conditions. The combination of lesion severity and extent supports objective quantification of clinical lesion burden, and owner-reported VAS scores add complementary welfare-relevant information. The simplified EqIS offers a practical alternative for routine use, with further evaluation needed to confirm performance across environments and geographic regions.

## Supplementary Material

Figure_S1_aalag132

Figure_S2_aalag132

Figure_S3_aalag132

Figure_S4_aalag132

Figure_S5_aalag132

Table_S1_aalag132

Table_S2_aalag132

Table_S3_aalag132

Supplementary_material_aalag132

## Off-label antimicrobial declaration

Authors declare no off-label use of antimicrobials.

## Institutional animal care and use committee or other approval declaration

The cantonal veterinary authorities approved the experiment, national number 33558 and 34147, leading canton Zurich ZH069/2021 and ZH202/2021, respectively.

## Appendix

### Supplementary Methods

This section provides detailed descriptions of scoring procedures, validation protocols, and statistical methods referenced in the main Materials and Methods section.

### Insect bite hypersensitivity (IBH) scoring system

#### Lesion severity score (Form 24)

Alopecia, blood/exudate, and crusts were classified as stand-alone lesions, whereas broken hair, scales, lichenification, and swelling were classified as non-stand-alone parameters. A lesion was defined by the presence of at least one stand-alone lesion or at least 2 non-stand-alone parameters within a body area. Each variable was scored according to its lesion-specific severity within the respective body area (0 = none; 1 = < 25%; 2 = 25%-<50%; 3 = 50%-<75%; 4 = ≥75%). These percentage categories refer to the degree of involvement of each individual lesion type and do not represent the overall proportion of the body area affected, which was assessed separately using the area score (Form 35). Scores were summed per body area and across all body areas to obtain the total lesion severity score (maximum 896 points).

To ensure consistent anatomical delineation and scoring reliability, predefined region-specific rules were applied. The ventral tail base was defined as the contact between the tail base and the genital region at rest. Lateral limb surfaces were excluded from medial limb scoring. The ventral crest of the mane was delineated using anatomical landmarks including the jugular groove, the scapula, and the poll.

All scorers received standardized training in application of the lesion severity score and used a photographic reference guide to ensure scoring consistency. The original scoring form is shown in Supplementary Figure S1A-F.

#### Area score (Form 35)

In contrast to the lesion severity score (Form 24), the area score (Form 35) reflects the overall proportion of the body area affected by any lesion, independent of lesion type. A body area was classified as affected according to the lesion presence rules defined for the lesion severity score (ie, at least one stand-alone lesion or 2 non-stand-alone variables), while the extend of all present variables accounted for the affected area, but excluding swelling and lichenification.

The proportion of affected skin within each body area was graded using a 6-point ordinal scale reflecting increasing involvement: 0 = absent (0%); 1 = mild (<10%); 1.5 = mild–moderate (10%-<25%); 2 = moderate (25%-<33%); 2.5 = moderate–severe (33%-<50%); and 3 = severe (≥50%).

Accordingly, an area score of 0 (“absent”) was assigned when no lesion presence criteria were not fulfilled, whereas “mild” involvement was assigned when at least one stand-alone lesion or 2 non-stand-alone variables were present and < 10% of the body area was affected.

Intermediate grades (1.5 or 2.5) were recorded by marking 2 adjacent categories on the form. Scores were recorded per body area and used as multiplicative weighting factors for calculation of the EqIS.

#### Equine IBH severity score (EqIS; Form 24 × 35)

The original scoring sheet is provided in Figure S3A-G.

#### Simplified EqIS (Form 37307)

The simplified EqIS scoring sheet (Form 37307) presented all predefined body areas in a condensed single-table format.

Lesion severity is graded on a 0-4 ordinal scale (0 = absent; 1 = mild; 2 = moderate; 3 = moderate–severe; 4 = severe) based on characteristic findings including broken hair, scales, alopecia, crusts, blood or exudate, swelling, or lichenification. The extent of involvement is recorded using categorical area factors (0%, <1%, 1%-<25%, 25%-<50%, ≥50%) corresponding to multiplicative weighting factors of 0, 0.5, 1.0, 1.25, and 1.5. For each body area, the lesion severity score is multiplied by the corresponding area factor to obtain a weighted regional score. The sum across all body areas yields the simplified EqIS.

The original scoring sheet is provided in Figure S4.

#### Owner-reported visual analog scale (VAS) scores

Owner-assessed VAS scores for overall IBH severity (I-VAS, Form 32) and pruritus intensity (P-VAS, Form 33) were recorded bi-weekly from mid-March until mid-October; the mean scores across the observation period were calculated for both instruments.

Each single VAS value consisted of a horizontal 10 cm line with anchors at both ends (0 = no clinical signs; 10 cm = maximum severity). Owners placed a vertical mark corresponding to the observed severity. The VAS score was defined as the distance in millimeters from the left end of the line to the mark.

Owners or caregivers familiar with the horse were permitted to perform the assessments. Standardized written instructions were provided before scoring. Completed forms were collected at scheduled visits throughout the observation period. VAS data were analyzed longitudinally and compared with clinician-assessed EqIS.

### Scoring procedures and score validation

#### Intraobserver reliability

For intraobserver reliability assessment, both the lesion severity score and the area score were recorded at each evaluation. Intraobserver agreement was evaluated using Pearson correlation coefficients and ICC, as described in the statistical analysis section.

#### Interobserver reliability

Interobserver scoring was performed under standardized field conditions during routine study visits. All scoring data were documented using standardized electronic case report forms (Prelude Dynamics, Austin, TX, USA) and archived according to good clinical practice requirements. Interobserver agreement was analyzed using Pearson correlation coefficients and ICCs, as described in the statistical analysis section.

#### Statistical analysis

All statistical analyses were conducted using GraphPad Prism (version 9.0; GraphPad Software, La Jolla, CA, USA) and R (irr package). All tests were 2-tailed, and significance was set at: **P* < .05, ***P* < .01, ****P* < .001, *****P* < .0001.

Intra- and interobserver reliability for the lesion severity score and the EqIS were assessed using ICC based on a 2-way mixed-effects model with absolute agreement. The ICCs were not calculated for the area score because the scale was not intended to function as an independent composite score, but rather as a multiplicative weighting factor within the EqIS. ICC(3,1) was applied to assess intraobserver reliability and ICC(3,k) for interobserver reliability. The ICC values are reported with 95% CI.

Agreement levels were categorized as excellent (ICC ≥ 0.90), good (ICC 0.75-0.89), moderate (ICC 0.50-0.74), and poor (ICC < 0.50).

## Abbreviations

IBH: insect bite hypersensitivity
EqIS: equine IBH severity score
