# Supplementary material for: Validation of an improved insect bite hypersensitivity severity score for allergic equine insect bite hypersensitivity in horses
Source: J Vet Intern Med. 2026 Jul 6;40(4):aalag132. doi: 10.1093/jvimsj/aalag132 (PMC13336633; doi:10.1093/jvimsj/aalag132)
Supplement: Figure_S1_aalag132 [file figure_s1_aalag132.pdf]

A)

STUDY ID

IBH LESION SCORE (FORM 24)

CASE ID	HORSE NAME	OWNER NAME	DATE (DDMMYY)

Points are given per summer eczema location or sublocation, respectively. The following parameters will be evaluated for each location and graded from 0 (absent) to 4 points. Broken hair, self-induced alopecia, blood/exudate, scales, and crusts are evaluated for the most severe lesion of each sublocation. Lichenification and swelling/bulges are evaluated as average for the whole sublocation. The sum gives sum per sublocation.

BODY AREA			BROKEN HAIR, IN % OF LENGTH BROKEN (most severe lesion)	SELF-INDUCED ALOPECIA (most severe lesion)	BLOOD / EXUDATE, AREA (most severe lesion)	SCALES area (%), ∅size (mm)	CRUSTS, AREA (most severe lesion)	LICHENIFICATIO N, AREA (whole location)	SWELLING / BULGES, AREA (whole location)	SUM
Head	Poll (Genick) & hairline forehead	1	☐0, absent<br>☐1, < 25%<br>☐2, 25%≤ x<50%<br>☐3, 50%≤x<75%<br>☐4, ≥75%	☐0, absent<br>☐1, < 25%<br>☐2, 25%≤ x<50%<br>☐3, 50%≤x<75%<br>☐4, ≥75%	☐0, absent<br>☐1, < 25%<br>☐2, 25%≤ x<50%<br>☐3, 50%≤x<75%<br>☐4, ≥75%	☐0, absent<br>☐1, <50%,≤2mm<br>☐2, <50%,>2mm<br>☐3, >50%,≤2mm<br>☐4, >50%,>2mm	☐0, absent<br>☐1, < 25%<br>☐2, 25%≤ x<50%<br>☐3, 50%≤x<75%<br>☐4, ≥75%	☐0, absent<br>☐1, < 25%<br>☐2, 25%≤ x<50%<br>☐3, 50%≤x<75%<br>☐4, ≥75%	☐0, absent<br>☐1, < 25%<br>☐2, 25%≤ x<50%<br>☐3, 50%≤x<75%<br>☐4, ≥75%	
	Left	2	☐0, absent<br>☐1, < 25%<br>☐2, 25%≤ x<50%<br>☐3, 50%≤x<75%<br>☐4, ≥75%	☐0, absent<br>☐1, < 25%<br>☐2, 25%≤ x<50%<br>☐3, 50%≤x<75%<br>☐4, ≥75%	☐0, absent<br>☐1, < 25%<br>☐2, 25%≤ x<50%<br>☐3, 50%≤x<75%<br>☐4, ≥75%	☐0, absent<br>☐1, <50%,≤2mm<br>☐2, <50%,>2mm<br>☐3, >50%,≤2mm<br>☐4, >50%,>2mm	☐0, absent<br>☐1, < 25%<br>☐2, 25%≤ x<50%<br>☐3, 50%≤x<75%<br>☐4, ≥75%	☐0, absent<br>☐1, < 25%<br>☐2, 25%≤ x<50%<br>☐3, 50%≤x<75%<br>☐4, ≥75%	☐0, absent<br>☐1, < 25%<br>☐2, 25%≤ x<50%<br>☐3, 50%≤x<75%<br>☐4, ≥75%	
	Right	3	☐0, absent<br>☐1, < 25%<br>☐2, 25%≤ x<50%<br>☐3, 50%≤x<75%<br>☐4, ≥75%	☐0, absent<br>☐1, < 25%<br>☐2, 25%≤ x<50%<br>☐3, 50%≤x<75%<br>☐4, ≥75%	☐0, absent<br>☐1, < 25%<br>☐2, 25%≤ x<50%<br>☐3, 50%≤x<75%<br>☐4, ≥75%	☐0, absent<br>☐1, <50%,≤2mm<br>☐2, <50%,>2mm<br>☐3, >50%,≤2mm<br>☐4, >50%,>2mm	☐0, absent<br>☐1, < 25%<br>☐2, 25%≤ x<50%<br>☐3, 50%≤x<75%<br>☐4, ≥75%	☐0, absent<br>☐1, < 25%<br>☐2, 25%≤ x<50%<br>☐3, 50%≤x<75%<br>☐4, ≥75%	☐0, absent<br>☐1, < 25%<br>☐2, 25%≤ x<50%<br>☐3, 50%≤x<75%<br>☐4, ≥75%	
	Ventral	4	☐0, absent<br>☐1, < 25%<br>☐2, 25%≤ x<50%<br>☐3, 50%≤x<75%<br>☐4, ≥75%	☐0, absent<br>☐1, < 25%<br>☐2, 25%≤ x<50%<br>☐3, 50%≤x<75%<br>☐4, ≥75%	☐0, absent<br>☐1, < 25%<br>☐2, 25%≤ x<50%<br>☐3, 50%≤x<75%<br>☐4, ≥75%	☐0, absent<br>☐1, <50%,≤2mm<br>☐2, <50%,>2mm<br>☐3, >50%,≤2mm<br>☐4, >50%,>2mm	☐0, absent<br>☐1, < 25%<br>☐2, 25%≤ x<50%<br>☐3, 50%≤x<75%<br>☐4, ≥75%	☐0, absent<br>☐1, < 25%<br>☐2, 25%≤ x<50%<br>☐3, 50%≤x<75%<br>☐4, ≥75%	☐0, absent<br>☐1, < 25%<br>☐2, 25%≤ x<50%<br>☐3, 50%≤x<75%<br>☐4, ≥75%	

B)

CASE ID	HORSE NAME	OWNER NAME	DATE (DDMMYY)

BODY AREA				BROKEN HAIR, IN % OF LENGTH BROKEN (most severe lesion)	SELF-INDUCED ALOPECIA (most severe lesion)	BLOOD / EXUDATE, AREA (most severe lesion)	SCALES (most severe lesion) area (%), ∅size (mm)	CRUSTS, AREA (most severe lesion)	LICHENIFICATION, AREA (whole location)	SWELLING / BULGES, AREA (whole location)	SUM
Ear	Left	Convex	5	☐0, absent<br>☐1, < 25%<br>☐2, 25%≤ x<50%<br>☐3, 50%≤x<75%<br>☐4, ≥75%	☐0, absent<br>☐1, < 25%<br>☐2, 25%≤ x<50%<br>☐3, 50%≤x<75%<br>☐4, ≥75%	☐0, absent<br>☐1, < 25%<br>☐2, 25%≤ x<50%<br>☐3, 50%≤x<75%<br>☐4, ≥75%	☐0, absent<br>☐1, <50%,≤2mm<br>☐2, <50%,>2mm<br>☐3, >50%,≤2mm<br>☐4, >50%,>2mm	☐0, absent<br>☐1, < 25%<br>☐2, 25%≤ x<50%<br>☐3, 50%≤x<75%<br>☐4, ≥75%	☐0, absent<br>☐1, < 25%<br>☐2, 25%≤ x<50%<br>☐3, 50%≤x<75%<br>☐4, ≥75%	☐0, absent<br>☐1, < 25%<br>☐2, 25%≤ x<50%<br>☐3, 50%≤x<75%<br>☐4, ≥75%	
		Concave	6	☐0, absent<br>☐1, < 25%<br>☐2, 25%≤ x<50%<br>☐3, 50%≤x<75%<br>☐4, ≥75%	☐0, absent<br>☐1, < 25%<br>☐2, 25%≤ x<50%<br>☐3, 50%≤x<75%<br>☐4, ≥75%	☐0, absent<br>☐1, < 25%<br>☐2, 25%≤ x<50%<br>☐3, 50%≤x<75%<br>☐4, ≥75%	☐0, absent<br>☐1, <50%,≤2mm<br>☐2, <50%,>2mm<br>☐3, >50%,≤2mm<br>☐4, >50%,>2mm	☐0, absent<br>☐1, < 25%<br>☐2, 25%≤ x<50%<br>☐3, 50%≤x<75%<br>☐4, ≥75%	☐0, absent<br>☐1, < 25%<br>☐2, 25%≤ x<50%<br>☐3, 50%≤x<75%<br>☐4, ≥75%	☐0, absent<br>☐1, < 25%<br>☐2, 25%≤ x<50%<br>☐3, 50%≤x<75%<br>☐4, ≥75%	
	Right	Convex	7	☐0, absent<br>☐1, < 25%<br>☐2, 25%≤ x<50%<br>☐3, 50%≤x<75%<br>☐4, ≥75%	☐0, absent<br>☐1, < 25%<br>☐2, 25%≤ x<50%<br>☐3, 50%≤x<75%<br>☐4, ≥75%	☐0, absent<br>☐1, < 25%<br>☐2, 25%≤ x<50%<br>☐3, 50%≤x<75%<br>☐4, ≥75%	☐0, absent<br>☐1, <50%,≤2mm<br>☐2, <50%,>2mm<br>☐3, >50%,≤2mm<br>☐4, >50%,>2mm	☐0, absent<br>☐1, < 25%<br>☐2, 25%≤ x<50%<br>☐3, 50%≤x<75%<br>☐4, ≥75%	☐0, absent<br>☐1, < 25%<br>☐2, 25%≤ x<50%<br>☐3, 50%≤x<75%<br>☐4, ≥75%	☐0, absent<br>☐1, < 25%<br>☐2, 25%≤ x<50%<br>☐3, 50%≤x<75%<br>☐4, ≥75%	
		Concave	8	☐0, absent<br>☐1, < 25%<br>☐2, 25%≤ x<50%<br>☐3, 50%≤x<75%<br>☐4, ≥75%	☐0, absent<br>☐1, < 25%<br>☐2, 25%≤ x<50%<br>☐3, 50%≤x<75%<br>☐4, ≥75%	☐0, absent<br>☐1, < 25%<br>☐2, 25%≤ x<50%<br>☐3, 50%≤x<75%<br>☐4, ≥75%	☐0, absent<br>☐1, <50%,≤2mm<br>☐2, <50%,>2mm<br>☐3, >50%,≤2mm<br>☐4, >50%,>2mm	☐0, absent<br>☐1, < 25%<br>☐2, 25%≤ x<50%<br>☐3, 50%≤x<75%<br>☐4, ≥75%	☐0, absent<br>☐1, < 25%<br>☐2, 25%≤ x<50%<br>☐3, 50%≤x<75%<br>☐4, ≥75%	☐0, absent<br>☐1, < 25%<br>☐2, 25%≤ x<50%<br>☐3, 50%≤x<75%<br>☐4, ≥75%	

C)

CASE ID	HORSE NAME	OWNER NAME	DATE (DDMMYY)

**BODY AREA**				**BROKEN HAIR, IN % OF LENGTH BROKEN (most severe lesion)**	**SELF-INDUCED ALOPECIA (most severe lesion)**	**BLOOD / EXUDATE, AREA (most severe lesion)**	**SCALES (most severe lesion) area (%), ∅size (mm)**	**CRUSTS, AREA (most severe lesion)**	**LICHENIFICATIO N, AREA (whole location)**	**SWELLING / BULGES, AREA (whole location)**	**SUM**
Mane / Neck	1/3 Crest cranial		9	☐0, absent<br>☐1, < 25%<br>☐2, 25%≤ x<50%<br>☐3, 50%≤x<75%<br>☐4, ≥75%	☐0, absent<br>☐1, < 25%<br>☐2, 25%≤ x<50%<br>☐3, 50%≤x<75%<br>☐4, ≥75%	☐0, absent<br>☐1, < 25%<br>☐2, 25%≤ x<50%<br>☐3, 50%≤x<75%<br>☐4, ≥75%	☐0, absent<br>☐1, <50%,≤2mm<br>☐2, <50%,>2mm<br>☐3, >50%,≤2mm<br>☐4, >50%,>2mm	☐0, absent<br>☐1, < 25%<br>☐2, 25%≤ x<50%<br>☐3, 50%≤x<75%<br>☐4, ≥75%	☐0, absent<br>☐1, < 25%<br>☐2, 25%≤ x<50%<br>☐3, 50%≤x<75%<br>☐4, ≥75%	☐0, absent<br>☐1, < 25%<br>☐2, 25%≤ x<50%<br>☐3, 50%≤x<75%<br>☐4, ≥75%	
	1/3 Crest middle		10	☐0, absent<br>☐1, < 25%<br>☐2, 25%≤ x<50%<br>☐3, 50%≤x<75%<br>☐4, ≥75%	☐0, absent<br>☐1, < 25%<br>☐2, 25%≤ x<50%<br>☐3, 50%≤x<75%<br>☐4, ≥75%	☐0, absent<br>☐1, < 25%<br>☐2, 25%≤ x<50%<br>☐3, 50%≤x<75%<br>☐4, ≥75%	☐0, absent<br>☐1, <50%,≤2mm<br>☐2, <50%,>2mm<br>☐3, >50%,≤2mm<br>☐4, >50%,>2mm	☐0, absent<br>☐1, < 25%<br>☐2, 25%≤ x<50%<br>☐3, 50%≤x<75%<br>☐4, ≥75%	☐0, absent<br>☐1, < 25%<br>☐2, 25%≤ x<50%<br>☐3, 50%≤x<75%<br>☐4, ≥75%	☐0, absent<br>☐1, < 25%<br>☐2, 25%≤ x<50%<br>☐3, 50%≤x<75%<br>☐4, ≥75%	
	1/3 Crest caudal		11	☐0, absent<br>☐1, < 25%<br>☐2, 25%≤ x<50%<br>☐3, 50%≤x<75%<br>☐4, ≥75%	☐0, absent<br>☐1, < 25%<br>☐2, 25%≤ x<50%<br>☐3, 50%≤x<75%<br>☐4, ≥75%	☐0, absent<br>☐1, < 25%<br>☐2, 25%≤ x<50%<br>☐3, 50%≤x<75%<br>☐4, ≥75%	☐0, absent<br>☐1, <50%,≤2mm<br>☐2, <50%,>2mm<br>☐3, >50%,≤2mm<br>☐4, >50%,>2mm	☐0, absent<br>☐1, < 25%<br>☐2, 25%≤ x<50%<br>☐3, 50%≤x<75%<br>☐4, ≥75%	☐0, absent<br>☐1, < 25%<br>☐2, 25%≤ x<50%<br>☐3, 50%≤x<75%<br>☐4, ≥75%	☐0, absent<br>☐1, < 25%<br>☐2, 25%≤ x<50%<br>☐3, 50%≤x<75%<br>☐4, ≥75%	
	Left	Crest ventral	12	☐0, absent<br>☐1, < 25%<br>☐2, 25%≤ x<50%<br>☐3, 50%≤x<75%<br>☐4, ≥75%	☐0, absent<br>☐1, < 25%<br>☐2, 25%≤ x<50%<br>☐3, 50%≤x<75%<br>☐4, ≥75%	☐0, absent<br>☐1, < 25%<br>☐2, 25%≤ x<50%<br>☐3, 50%≤x<75%<br>☐4, ≥75%	☐0, absent<br>☐1, <50%,≤2mm<br>☐2, <50%,>2mm<br>☐3, >50%,≤2mm<br>☐4, >50%,>2mm	☐0, absent<br>☐1, < 25%<br>☐2, 25%≤ x<50%<br>☐3, 50%≤x<75%<br>☐4, ≥75%	☐0, absent<br>☐1, < 25%<br>☐2, 25%≤ x<50%<br>☐3, 50%≤x<75%<br>☐4, ≥75%	☐0, absent<br>☐1, < 25%<br>☐2, 25%≤ x<50%<br>☐3, 50%≤x<75%<br>☐4, ≥75%	
	Right	Crest ventral	13	☐0, absent<br>☐1, < 25%<br>☐2, 25%≤ x<50%<br>☐3, 50%≤x<75%<br>☐4, ≥75%	☐0, absent<br>☐1, < 25%<br>☐2, 25%≤ x<50%<br>☐3, 50%≤x<75%<br>☐4, ≥75%	☐0, absent<br>☐1, < 25%<br>☐2, 25%≤ x<50%<br>☐3, 50%≤x<75%<br>☐4, ≥75%	☐0, absent<br>☐1, <50%,≤2mm<br>☐2, <50%,>2mm<br>☐3, >50%,≤2mm<br>☐4, >50%,>2mm	☐0, absent<br>☐1, < 25%<br>☐2, 25%≤ x<50%<br>☐3, 50%≤x<75%<br>☐4, ≥75%	☐0, absent<br>☐1, < 25%<br>☐2, 25%≤ x<50%<br>☐3, 50%≤x<75%<br>☐4, ≥75%	☐0, absent<br>☐1, < 25%<br>☐2, 25%≤ x<50%<br>☐3, 50%≤x<75%<br>☐4, ≥75%	
Breast	cranial		14	☐0, absent<br>☐1, < 25%<br>☐2, 25%≤ x<50%<br>☐3, 50%≤x<75%<br>☐4, ≥75%	☐0, absent<br>☐1, < 25%<br>☐2, 25%≤ x<50%<br>☐3, 50%≤x<75%<br>☐4, ≥75%	☐0, absent<br>☐1, < 25%<br>☐2, 25%≤ x<50%<br>☐3, 50%≤x<75%<br>☐4, ≥75%	☐0, absent<br>☐1, <50%,≤2mm<br>☐2, <50%,>2mm<br>☐3, >50%,≤2mm<br>☐4, >50%,>2mm	☐0, absent<br>☐1, < 25%<br>☐2, 25%≤ x<50%<br>☐3, 50%≤x<75%<br>☐4, ≥75%	☐0, absent<br>☐1, < 25%<br>☐2, 25%≤ x<50%<br>☐3, 50%≤x<75%<br>☐4, ≥75%	☐0, absent<br>☐1, < 25%<br>☐2, 25%≤ x<50%<br>☐3, 50%≤x<75%<br>☐4, ≥75%	

D)

CASE ID	HORSE NAME	OWNER NAME	DATE (DDMMYY)

BODY AREA			BROKEN HAIR, IN % OF LENGTH BROKEN (most severe lesion)	SELF-INDUCED ALOPECIA (most severe lesion)	BLOOD / EXUDATE, AREA (most severe lesion)	SCALES (most severe lesion) area (%), ∅size (mm)	CRUSTS, AREA (most severe lesion)	LICHENIFICATIO N, AREA (whole location)	SWELLING / BULGES, AREA (whole location)	SUM
Axilla	Left	15	☐0, absent<br>☐1, < 25%<br>☐2, 25%≤ x<50%<br>☐3, 50%≤x<75%<br>☐4, ≥75%	☐0, absent<br>☐1, < 25%<br>☐2, 25%≤ x<50%<br>☐3, 50%≤x<75%<br>☐4, ≥75%	☐0, absent<br>☐1, < 25%<br>☐2, 25%≤ x<50%<br>☐3, 50%≤x<75%<br>☐4, ≥75%	☐0, absent<br>☐1, <50%,≤2mm<br>☐2, <50%,>2mm<br>☐3, >50%,≤2mm<br>☐4, >50%,>2mm	☐0, absent<br>☐1, < 25%<br>☐2, 25%≤ x<50%<br>☐3, 50%≤x<75%<br>☐4, ≥75%	☐0, absent<br>☐1, < 25%<br>☐2, 25%≤ x<50%<br>☐3, 50%≤x<75%<br>☐4, ≥75%	☐0, absent<br>☐1, < 25%<br>☐2, 25%≤ x<50%<br>☐3, 50%≤x<75%<br>☐4, ≥75%	
	Right	16	☐0, absent<br>☐1, < 25%<br>☐2, 25%≤ x<50%<br>☐3, 50%≤x<75%<br>☐4, ≥75%	☐0, absent<br>☐1, < 25%<br>☐2, 25%≤ x<50%<br>☐3, 50%≤x<75%<br>☐4, ≥75%	☐0, absent<br>☐1, < 25%<br>☐2, 25%≤ x<50%<br>☐3, 50%≤x<75%<br>☐4, ≥75%	☐0, absent<br>☐1, <50%,≤2mm<br>☐2, <50%,>2mm<br>☐3, >50%,≤2mm<br>☐4, >50%,>2mm	☐0, absent<br>☐1, < 25%<br>☐2, 25%≤ x<50%<br>☐3, 50%≤x<75%<br>☐4, ≥75%	☐0, absent<br>☐1, < 25%<br>☐2, 25%≤ x<50%<br>☐3, 50%≤x<75%<br>☐4, ≥75%	☐0, absent<br>☐1, < 25%<br>☐2, 25%≤ x<50%<br>☐3, 50%≤x<75%<br>☐4, ≥75%	
Ventral Midline	1/3 cranial	17	☐0, absent<br>☐1, < 25%<br>☐2, 25%≤ x<50%<br>☐3, 50%≤x<75%<br>☐4, ≥75%	☐0, absent<br>☐1, < 25%<br>☐2, 25%≤ x<50%<br>☐3, 50%≤x<75%<br>☐4, ≥75%	☐0, absent<br>☐1, < 25%<br>☐2, 25%≤ x<50%<br>☐3, 50%≤x<75%<br>☐4, ≥75%	☐0, absent<br>☐1, <50%,≤2mm<br>☐2, <50%,>2mm<br>☐3, >50%,≤2mm<br>☐4, >50%,>2mm	☐0, absent<br>☐1, < 25%<br>☐2, 25%≤ x<50%<br>☐3, 50%≤x<75%<br>☐4, ≥75%	☐0, absent<br>☐1, < 25%<br>☐2, 25%≤ x<50%<br>☐3, 50%≤x<75%<br>☐4, ≥75%	☐0, absent<br>☐1, < 25%<br>☐2, 25%≤ x<50%<br>☐3, 50%≤x<75%<br>☐4, ≥75%	
	1/3 middle	18	☐0, absent<br>☐1, < 25%<br>☐2, 25%≤ x<50%<br>☐3, 50%≤x<75%<br>☐4, ≥75%	☐0, absent<br>☐1, < 25%<br>☐2, 25%≤ x<50%<br>☐3, 50%≤x<75%<br>☐4, ≥75%	☐0, absent<br>☐1, < 25%<br>☐2, 25%≤ x<50%<br>☐3, 50%≤x<75%<br>☐4, ≥75%	☐0, absent<br>☐1, <50%,≤2mm<br>☐2, <50%,>2mm<br>☐3, >50%,≤2mm<br>☐4, >50%,>2mm	☐0, absent<br>☐1, < 25%<br>☐2, 25%≤ x<50%<br>☐3, 50%≤x<75%<br>☐4, ≥75%	☐0, absent<br>☐1, < 25%<br>☐2, 25%≤ x<50%<br>☐3, 50%≤x<75%<br>☐4, ≥75%	☐0, absent<br>☐1, < 25%<br>☐2, 25%≤ x<50%<br>☐3, 50%≤x<75%<br>☐4, ≥75%	
	1/3 caudal	19	☐0, absent<br>☐1, < 25%<br>☐2, 25%≤ x<50%<br>☐3, 50%≤x<75%<br>☐4, ≥75%	☐0, absent<br>☐1, < 25%<br>☐2, 25%≤ x<50%<br>☐3, 50%≤x<75%<br>☐4, ≥75%	☐0, absent<br>☐1, < 25%<br>☐2, 25%≤ x<50%<br>☐3, 50%≤x<75%<br>☐4, ≥75%	☐0, absent<br>☐1, <50%,≤2mm<br>☐2, <50%,>2mm<br>☐3, >50%,≤2mm<br>☐4, >50%,>2mm	☐0, absent<br>☐1, < 25%<br>☐2, 25%≤ x<50%<br>☐3, 50%≤x<75%<br>☐4, ≥75%	☐0, absent<br>☐1, < 25%<br>☐2, 25%≤ x<50%<br>☐3, 50%≤x<75%<br>☐4, ≥75%	☐0, absent<br>☐1, < 25%<br>☐2, 25%≤ x<50%<br>☐3, 50%≤x<75%<br>☐4, ≥75%	
	Prepuce / Udder	20	☐0, absent<br>☐1, < 25%<br>☐2, 25%≤ x<50%<br>☐3, 50%≤x<75%<br>☐4, ≥75%	☐0, absent<br>☐1, < 25%<br>☐2, 25%≤ x<50%<br>☐3, 50%≤x<75%<br>☐4, ≥75%	☐0, absent<br>☐1, < 25%<br>☐2, 25%≤ x<50%<br>☐3, 50%≤x<75%<br>☐4, ≥75%	☐0, absent<br>☐1, <50%,≤2mm<br>☐2, <50%,>2mm<br>☐3, >50%,≤2mm<br>☐4, >50%,>2mm	☐0, absent<br>☐1, < 25%<br>☐2, 25%≤ x<50%<br>☐3, 50%≤x<75%<br>☐4, ≥75%	☐0, absent<br>☐1, < 25%<br>☐2, 25%≤ x<50%<br>☐3, 50%≤x<75%<br>☐4, ≥75%	☐0, absent<br>☐1, < 25%<br>☐2, 25%≤ x<50%<br>☐3, 50%≤x<75%<br>☐4, ≥75%	

E)

CASE ID	HORSE NAME	OWNER NAME	DATE (DDMMYY)

BODY AREA			BROKEN HAIR, IN % OF LENGTH BROKEN (most severe lesion)	SELF-INDUCED ALOPECIA (most severe lesion)	BLOOD / EXUDATE, AREA (most severe lesion)	SCALES (most severe lesion) area (%), ∅size (mm)	CRUSTS, AREA (most severe lesion)	LICHENIFICATION, AREA (whole location)	SWELLING / BULGES, AREA (whole location)	SUM
Fore limb	Medial right & left	21	☐0, absent<br>☐1, < 25%<br>☐2, 25%≤ x<50%<br>☐3, 50%≤x<75%<br>☐4, ≥75%	☐0, absent<br>☐1, < 25%<br>☐2, 25%≤ x<50%<br>☐3, 50%≤x<75%<br>☐4, ≥75%	☐0, absent<br>☐1, < 25%<br>☐2, 25%≤ x<50%<br>☐3, 50%≤x<75%<br>☐4, ≥75%	☐0, absent<br>☐1, <50%,≤2mm<br>☐2, <50%,>2mm<br>☐3, >50%,≤2mm<br>☐4, >50%,>2mm	☐0, absent<br>☐1, < 25%<br>☐2, 25%≤ x<50%<br>☐3, 50%≤x<75%<br>☐4, ≥75%	☐0, absent<br>☐1, < 25%<br>☐2, 25%≤ x<50%<br>☐3, 50%≤x<75%<br>☐4, ≥75%	☐0, absent<br>☐1, < 25%<br>☐2, 25%≤ x<50%<br>☐3, 50%≤x<75%<br>☐4, ≥75%	
	Carpal joint	22	☐0, absent<br>☐1, < 25%<br>☐2, 25%≤ x<50%<br>☐3, 50%≤x<75%<br>☐4, ≥75%	☐0, absent<br>☐1, < 25%<br>☐2, 25%≤ x<50%<br>☐3, 50%≤x<75%<br>☐4, ≥75%	☐0, absent<br>☐1, < 25%<br>☐2, 25%≤ x<50%<br>☐3, 50%≤x<75%<br>☐4, ≥75%	☐0, absent<br>☐1, <50%,≤2mm<br>☐2, <50%,>2mm<br>☐3, >50%,≤2mm<br>☐4, >50%,>2mm	☐0, absent<br>☐1, < 25%<br>☐2, 25%≤ x<50%<br>☐3, 50%≤x<75%<br>☐4, ≥75%	☐0, absent<br>☐1, < 25%<br>☐2, 25%≤ x<50%<br>☐3, 50%≤x<75%<br>☐4, ≥75%	☐0, absent<br>☐1, < 25%<br>☐2, 25%≤ x<50%<br>☐3, 50%≤x<75%<br>☐4, ≥75%	
Hind limb	Medial right & left	23	☐0, absent<br>☐1, < 25%<br>☐2, 25%≤ x<50%<br>☐3, 50%≤x<75%<br>☐4, ≥75%	☐0, absent<br>☐1, < 25%<br>☐2, 25%≤ x<50%<br>☐3, 50%≤x<75%<br>☐4, ≥75%	☐0, absent<br>☐1, < 25%<br>☐2, 25%≤ x<50%<br>☐3, 50%≤x<75%<br>☐4, ≥75%	☐0, absent<br>☐1, <50%,≤2mm<br>☐2, <50%,>2mm<br>☐3, >50%,≤2mm<br>☐4, >50%,>2mm	☐0, absent<br>☐1, < 25%<br>☐2, 25%≤ x<50%<br>☐3, 50%≤x<75%<br>☐4, ≥75%	☐0, absent<br>☐1, < 25%<br>☐2, 25%≤ x<50%<br>☐3, 50%≤x<75%<br>☐4, ≥75%	☐0, absent<br>☐1, < 25%<br>☐2, 25%≤ x<50%<br>☐3, 50%≤x<75%<br>☐4, ≥75%	
	Tarsal joint	24	☐0, absent<br>☐1, < 25%<br>☐2, 25%≤ x<50%<br>☐3, 50%≤x<75%<br>☐4, ≥75%	☐0, absent<br>☐1, < 25%<br>☐2, 25%≤ x<50%<br>☐3, 50%≤x<75%<br>☐4, ≥75%	☐0, absent<br>☐1, < 25%<br>☐2, 25%≤ x<50%<br>☐3, 50%≤x<75%<br>☐4, ≥75%	☐0, absent<br>☐1, <50%,≤2mm<br>☐2, <50%,>2mm<br>☐3, >50%,≤2mm<br>☐4, >50%,>2mm	☐0, absent<br>☐1, < 25%<br>☐2, 25%≤ x<50%<br>☐3, 50%≤x<75%<br>☐4, ≥75%	☐0, absent<br>☐1, < 25%<br>☐2, 25%≤ x<50%<br>☐3, 50%≤x<75%<br>☐4, ≥75%	☐0, absent<br>☐1, < 25%<br>☐2, 25%≤ x<50%<br>☐3, 50%≤x<75%<br>☐4, ≥75%	
Flank	Left	25	☐0, absent<br>☐1, < 25%<br>☐2, 25%≤ x<50%<br>☐3, 50%≤x<75%<br>☐4, ≥75%	☐0, absent<br>☐1, < 25%<br>☐2, 25%≤ x<50%<br>☐3, 50%≤x<75%<br>☐4, ≥75%	☐0, absent<br>☐1, < 25%<br>☐2, 25%≤ x<50%<br>☐3, 50%≤x<75%<br>☐4, ≥75%	☐0, absent<br>☐1, <50%,≤2mm<br>☐2, <50%,>2mm<br>☐3, >50%,≤2mm<br>☐4, >50%,>2mm	☐0, absent<br>☐1, < 25%<br>☐2, 25%≤ x<50%<br>☐3, 50%≤x<75%<br>☐4, ≥75%	☐0, absent<br>☐1, < 25%<br>☐2, 25%≤ x<50%<br>☐3, 50%≤x<75%<br>☐4, ≥75%	☐0, absent<br>☐1, < 25%<br>☐2, 25%≤ x<50%<br>☐3, 50%≤x<75%<br>☐4, ≥75%	
	Right	26	☐0, absent<br>☐1, < 25%<br>☐2, 25%≤ x<50%<br>☐3, 50%≤x<75%<br>☐4, ≥75%	☐0, absent<br>☐1, < 25%<br>☐2, 25%≤ x<50%<br>☐3, 50%≤x<75%<br>☐4, ≥75%	☐0, absent<br>☐1, < 25%<br>☐2, 25%≤ x<50%<br>☐3, 50%≤x<75%<br>☐4, ≥75%	☐0, absent<br>☐1, <50%,≤2mm<br>☐2, <50%,>2mm<br>☐3, >50%,≤2mm<br>☐4, >50%,>2mm	☐0, absent<br>☐1, < 25%<br>☐2, 25%≤ x<50%<br>☐3, 50%≤x<75%<br>☐4, ≥75%	☐0, absent<br>☐1, < 25%<br>☐2, 25%≤ x<50%<br>☐3, 50%≤x<75%<br>☐4, ≥75%	☐0, absent<br>☐1, < 25%<br>☐2, 25%≤ x<50%<br>☐3, 50%≤x<75%<br>☐4, ≥75%	

F)

CASE ID	HORSE NAME	OWNER NAME	DATE (DDMMYY)

BODY AREA			BROKEN HAIR, IN % OF LENGTH BROKEN (most severe lesion)	SELF-INDUCED ALOPECIA (most severe lesion)	BLOOD / EXUDATE, AREA (most severe lesion)	SCALES (most severe lesion) area (%), ∅size (mm)	CRUSTS, AREA (most severe lesion)	LICHENIFICATIO N, AREA (whole location)	SWELLING / BULGES, AREA (whole location)	SUM
Croup	Dorsal	27	☐0, absent<br>☐1, < 25%<br>☐2, 25%≤ x<50%<br>☐3, 50%≤x<75%<br>☐4, ≥75%	☐0, absent<br>☐1, < 25%<br>☐2, 25%≤ x<50%<br>☐3, 50%≤x<75%<br>☐4, ≥75%	☐0, absent<br>☐1, < 25%<br>☐2, 25%≤ x<50%<br>☐3, 50%≤x<75%<br>☐4, ≥75%	☐0, absent<br>☐1, <50%,≤2mm<br>☐2, <50%,>2mm<br>☐3, >50%,≤2mm<br>☐4, >50%,>2mm	☐0, absent<br>☐1, < 25%<br>☐2, 25%≤ x<50%<br>☐3, 50%≤x<75%<br>☐4, ≥75%	☐0, absent<br>☐1, < 25%<br>☐2, 25%≤ x<50%<br>☐3, 50%≤x<75%<br>☐4, ≥75%	☐0, absent<br>☐1, < 25%<br>☐2, 25%≤ x<50%<br>☐3, 50%≤x<75%<br>☐4, ≥75%	
	Caudal	28	☐0, absent<br>☐1, < 25%<br>☐2, 25%≤ x<50%<br>☐3, 50%≤x<75%<br>☐4, ≥75%	☐0, absent<br>☐1, < 25%<br>☐2, 25%≤ x<50%<br>☐3, 50%≤x<75%<br>☐4, ≥75%	☐0, absent<br>☐1, < 25%<br>☐2, 25%≤ x<50%<br>☐3, 50%≤x<75%<br>☐4, ≥75%	☐0, absent<br>☐1, <50%,≤2mm<br>☐2, <50%,>2mm<br>☐3, >50%,≤2mm<br>☐4, >50%,>2mm	☐0, absent<br>☐1, < 25%<br>☐2, 25%≤ x<50%<br>☐3, 50%≤x<75%<br>☐4, ≥75%	☐0, absent<br>☐1, < 25%<br>☐2, 25%≤ x<50%<br>☐3, 50%≤x<75%<br>☐4, ≥75%	☐0, absent<br>☐1, < 25%<br>☐2, 25%≤ x<50%<br>☐3, 50%≤x<75%<br>☐4, ≥75%	
Tail	1/3 proximal	29	☐0, absent<br>☐1, < 25%<br>☐2, 25%≤ x<50%<br>☐3, 50%≤x<75%<br>☐4, ≥75%	☐0, absent<br>☐1, < 25%<br>☐2, 25%≤ x<50%<br>☐3, 50%≤x<75%<br>☐4, ≥75%	☐0, absent<br>☐1, < 25%<br>☐2, 25%≤ x<50%<br>☐3, 50%≤x<75%<br>☐4, ≥75%	☐0, absent<br>☐1, <50%,≤2mm<br>☐2, <50%,>2mm<br>☐3, >50%,≤2mm<br>☐4, >50%,>2mm	☐0, absent<br>☐1, < 25%<br>☐2, 25%≤ x<50%<br>☐3, 50%≤x<75%<br>☐4, ≥75%	☐0, absent<br>☐1, < 25%<br>☐2, 25%≤ x<50%<br>☐3, 50%≤x<75%<br>☐4, ≥75%	☐0, absent<br>☐1, < 25%<br>☐2, 25%≤ x<50%<br>☐3, 50%≤x<75%<br>☐4, ≥75%	
	1/3 middle	30	☐0, absent<br>☐1, < 25%<br>☐2, 25%≤ x<50%<br>☐3, 50%≤x<75%<br>☐4, ≥75%	☐0, absent<br>☐1, < 25%<br>☐2, 25%≤ x<50%<br>☐3, 50%≤x<75%<br>☐4, ≥75%	☐0, absent<br>☐1, < 25%<br>☐2, 25%≤ x<50%<br>☐3, 50%≤x<75%<br>☐4, ≥75%	☐0, absent<br>☐1, <50%,≤2mm<br>☐2, <50%,>2mm<br>☐3, >50%,≤2mm<br>☐4, >50%,>2mm	☐0, absent<br>☐1, < 25%<br>☐2, 25%≤ x<50%<br>☐3, 50%≤x<75%<br>☐4, ≥75%	☐0, absent<br>☐1, < 25%<br>☐2, 25%≤ x<50%<br>☐3, 50%≤x<75%<br>☐4, ≥75%	☐0, absent<br>☐1, < 25%<br>☐2, 25%≤ x<50%<br>☐3, 50%≤x<75%<br>☐4, ≥75%	
	1/3 distal	31	☐0, absent<br>☐1, < 25%<br>☐2, 25%≤ x<50%<br>☐3, 50%≤x<75%<br>☐4, ≥75%	☐0, absent<br>☐1, < 25%<br>☐2, 25%≤ x<50%<br>☐3, 50%≤x<75%<br>☐4, ≥75%	☐0, absent<br>☐1, < 25%<br>☐2, 25%≤ x<50%<br>☐3, 50%≤x<75%<br>☐4, ≥75%	☐0, absent<br>☐1, <50%,≤2mm<br>☐2, <50%,>2mm<br>☐3, >50%,≤2mm<br>☐4, >50%,>2mm	☐0, absent<br>☐1, < 25%<br>☐2, 25%≤ x<50%<br>☐3, 50%≤x<75%<br>☐4, ≥75%	☐0, absent<br>☐1, < 25%<br>☐2, 25%≤ x<50%<br>☐3, 50%≤x<75%<br>☐4, ≥75%	☐0, absent<br>☐1, < 25%<br>☐2, 25%≤ x<50%<br>☐3, 50%≤x<75%<br>☐4, ≥75%	
	Ventral surface of tail	32	☐0, absent<br>☐1, < 25%<br>☐2, 25%≤ x<50%<br>☐3, 50%≤x<75%<br>☐4, ≥75%	☐0, absent<br>☐1, < 25%<br>☐2, 25%≤ x<50%<br>☐3, 50%≤x<75%<br>☐4, ≥75%	☐0, absent<br>☐1, < 25%<br>☐2, 25%≤ x<50%<br>☐3, 50%≤x<75%<br>☐4, ≥75%	☐0, absent<br>☐1, <50%,≤2mm<br>☐2, <50%,>2mm<br>☐3, >50%,≤2mm<br>☐4, >50%,>2mm	☐0, absent<br>☐1, < 25%<br>☐2, 25%≤ x<50%<br>☐3, 50%≤x<75%<br>☐4, ≥75%	☐0, absent<br>☐1, < 25%<br>☐2, 25%≤ x<50%<br>☐3, 50%≤x<75%<br>☐4, ≥75%	☐0, absent<br>☐1, < 25%<br>☐2, 25%≤ x<50%<br>☐3, 50%≤x<75%<br>☐4, ≥75%	
**TOTAL SCORE** (896 maximum)										

Recorded by (initials)		DATE RECORDED (DDMMYY)		INV. REVIEW (initials)		DATE RECORDED (DDMMYY)	
