# Supplementary material for: Validation of an improved insect bite hypersensitivity severity score for allergic equine insect bite hypersensitivity in horses
Source: J Vet Intern Med. 2026 Jul 6;40(4):aalag132. doi: 10.1093/jvimsj/aalag132 (PMC13336633; doi:10.1093/jvimsj/aalag132)
Supplement: Figure_S2_aalag132 [file figure_s2_aalag132.pdf]

STUDY ID

IBH SEVERITY (FORM 35)

CASE ID	HORSE NAME	OWNER NAME	DATE (DDMMYY)

Points are given per summer eczema location, according to photo examples ANNEX FORM 35. The following parameters will be evaluated for each location and graded from 0 (absent), 1 (mild), moderate (2) to 3 points (severe). In case observer wants to give an intermediate grade, two boxes will be clicked.

BODY AREA				SEVERITY ASSESSMENT	SUM
Head	Poll (Genick) & hairline forehead		1	☐0, absent<br>☐1, mild<br>☐2, moderate<br>☐3, severe	
	Left		2	☐0, absent<br>☐1, mild<br>☐2, moderate<br>☐3, severe	
	Right		3	☐0, absent<br>☐1, mild<br>☐2, moderate<br>☐3, severe	
	Ventral		4	☐0, absent<br>☐1, mild<br>☐2, moderate<br>☐3, severe	
Ear	Left	Convex	5	☐0, absent<br>☐1, mild<br>☐2, moderate<br>☐3, severe	
		Concave	6	☐0, absent<br>☐1, mild<br>☐2, moderate<br>☐3, severe	
	Right	Convex	7	☐0, absent<br>☐1, mild<br>☐2, moderate<br>☐3, severe	
		Concave	8	☐0, absent<br>☐1, mild<br>☐2, moderate<br>☐3, severe	
Mane / Neck	1/3 Crest cranial		9	☐0, absent<br>☐1, mild<br>☐2, moderate<br>☐3, severe	
	1/3 Crest middle		10	☐0, absent<br>☐1, mild<br>☐2, moderate<br>☐3, severe	
	1/3 Crest caudal		11	☐0, absent<br>☐1, mild<br>☐2, moderate<br>☐3, severe	
	Left	Crest ventral	12	☐0, absent<br>☐1, mild<br>☐2, moderate<br>☐3, severe	
	Right	Crest ventral	13	☐0, absent<br>☐1, mild<br>☐2, moderate<br>☐3, severe	
Breast	cranial		14	☐0, absent<br>☐1, mild<br>☐2, moderate<br>☐3, severe	
Axilla	Left		15	☐0, absent<br>☐1, mild<br>☐2, moderate<br>☐3, severe	
	Right		16	☐0, absent<br>☐1, mild<br>☐2, moderate<br>☐3, severe	
Ventral Midline	1/3 cranial		17	☐0, absent<br>☐1, mild<br>☐2, moderate<br>☐3, severe	
	1/3 middle		18	☐0, absent<br>☐1, mild<br>☐2, moderate<br>☐3, severe	
	1/3 caudal		19	☐0, absent<br>☐1, mild<br>☐2, moderate<br>☐3, severe	
	Prepuce / Udder		20	☐0, absent<br>☐1, mild<br>☐2, moderate<br>☐3, severe	
Fore limb	Medial right & left		21	☐0, absent<br>☐1, mild<br>☐2, moderate<br>☐3, severe	
	Carpal joint		22	☐0, absent<br>☐1, mild<br>☐2, moderate<br>☐3, severe	
Hind limb	Medial right & left		23	☐0, absent<br>☐1, mild<br>☐2, moderate<br>☐3, severe	
	Tarsal joint		24	☐0, absent<br>☐1, mild<br>☐2, moderate<br>☐3, severe	
Flank	Left		25	☐0, absent<br>☐1, mild<br>☐2, moderate<br>☐3, severe	
	Right		26	☐0, absent<br>☐1, mild<br>☐2, moderate<br>☐3, severe	
Croup	Dorsal		27	☐0, absent<br>☐1, mild<br>☐2, moderate<br>☐3, severe	
	Caudal		28	☐0, absent<br>☐1, mild<br>☐2, moderate<br>☐3, severe	
Tail	1/3 proximal		29	☐0, absent<br>☐1, mild<br>☐2, moderate<br>☐3, severe	
	1/3 middle		30	☐0, absent<br>☐1, mild<br>☐2, moderate<br>☐3, severe	
	1/3 distal		31	☐0, absent<br>☐1, mild<br>☐2, moderate<br>☐3, severe	
	Ventral surface of tail		32	☐0, absent<br>☐1, mild<br>☐2, moderate<br>☐3, severe	
**SUM**					

**EXAMINER (initials)**		**DATE RECORDED (DDMMYY)**	
**INV. REVIEW (initials)**		**DATE RECORDED (DDMMYY)**	
