# Supplementary material for: Validation of an improved insect bite hypersensitivity severity score for allergic equine insect bite hypersensitivity in horses
Source: J Vet Intern Med. 2026 Jul 6;40(4):aalag132. doi: 10.1093/jvimsj/aalag132 (PMC13336633; doi:10.1093/jvimsj/aalag132)
Supplement: Figure_S4_aalag132 [file figure_s4_aalag132.pdf]

SCORE SHEET INSECT BITE HYPERSENSITIVITY LESION SEVERITY

CASE ID	HORSE NAME

**INV. REVIEW (initials)**	
**DATE RECORDED (DDMMYY)**	

Characteristics of lesions: broken hair, scales, alopecia, blood/exudate, crust, swelling/lichenifiction
Points per body site: 0, absent (no lesions); 1, mild (broken hair, scales); 2, moderate (alopecia, crusts); 3, moderate-severe (blood/excudate); 4, severe (blood/excudate, swelling/lichenification)
Area affected: 0, 0%; a, < 1%; b (factor x0.5), 1% ≤ x < 25% (factor x1)*; c,* 25% ≤ x < 50% (factor x1.25); d, ≤ 50% (factor x1.5)

**BODY AREA**	**V#1, DATE:** ______________	**V#2, DATE:** ______________	**V#3, DATE:** ______________	**V#4, DATE:** ______________	**V#5, DATE:** ______________	**V#6, DATE:** ______________	**V#7, DATE:** ______________
Head	☐0 ☐1 ☐2 ☐3 ☐4<br>☐0 ☐a ☐b ☐c ☐d	☐0 ☐1 ☐2 ☐3 ☐4<br>☐0 ☐a ☐b ☐c ☐d	☐0 ☐1 ☐2 ☐3 ☐4<br>☐0 ☐a ☐b ☐c ☐d	☐0 ☐1 ☐2 ☐3 ☐4<br>☐0 ☐a ☐b ☐c ☐d	☐0 ☐1 ☐2 ☐3 ☐4<br>☐0 ☐a ☐b ☐c ☐d	☐0 ☐1 ☐2 ☐3 ☐4<br>☐0 ☐a ☐b ☐c ☐d	☐0 ☐1 ☐2 ☐3 ☐4<br>☐0 ☐a ☐b ☐c ☐d
Ears	☐0 ☐1 ☐2 ☐3 ☐4<br>☐0 ☐a ☐b ☐c ☐d	☐0 ☐1 ☐2 ☐3 ☐4<br>☐0 ☐a ☐b ☐c ☐d	☐0 ☐1 ☐2 ☐3 ☐4<br>☐0 ☐a ☐b ☐c ☐d	☐0 ☐1 ☐2 ☐3 ☐4<br>☐0 ☐a ☐b ☐c ☐d	☐0 ☐1 ☐2 ☐3 ☐4<br>☐0 ☐a ☐b ☐c ☐d	☐0 ☐1 ☐2 ☐3 ☐4<br>☐0 ☐a ☐b ☐c ☐d	☐0 ☐1 ☐2 ☐3 ☐4<br>☐0 ☐a ☐b ☐c ☐d
Mane 1/3 crest cranial	☐0 ☐1 ☐2 ☐3 ☐4<br>☐0 ☐a ☐b ☐c ☐d	☐0 ☐1 ☐2 ☐3 ☐4<br>☐0 ☐a ☐b ☐c ☐d	☐0 ☐1 ☐2 ☐3 ☐4<br>☐0 ☐a ☐b ☐c ☐d	☐0 ☐1 ☐2 ☐3 ☐4<br>☐0 ☐a ☐b ☐c ☐d	☐0 ☐1 ☐2 ☐3 ☐4<br>☐0 ☐a ☐b ☐c ☐d	☐0 ☐1 ☐2 ☐3 ☐4<br>☐0 ☐a ☐b ☐c ☐d	☐0 ☐1 ☐2 ☐3 ☐4<br>☐0 ☐a ☐b ☐c ☐d
Mane 1/3 crest middle	☐0 ☐1 ☐2 ☐3 ☐4<br>☐0 ☐a ☐b ☐c ☐d	☐0 ☐1 ☐2 ☐3 ☐4<br>☐0 ☐a ☐b ☐c ☐d	☐0 ☐1 ☐2 ☐3 ☐4<br>☐0 ☐a ☐b ☐c ☐d	☐0 ☐1 ☐2 ☐3 ☐4<br>☐0 ☐a ☐b ☐c ☐d	☐0 ☐1 ☐2 ☐3 ☐4<br>☐0 ☐a ☐b ☐c ☐d	☐0 ☐1 ☐2 ☐3 ☐4<br>☐0 ☐a ☐b ☐c ☐d	☐0 ☐1 ☐2 ☐3 ☐4<br>☐0 ☐a ☐b ☐c ☐d
Mane 1/3 crest caudal	☐0 ☐1 ☐2 ☐3 ☐4<br>☐0 ☐a ☐b ☐c ☐d	☐0 ☐1 ☐2 ☐3 ☐4<br>☐0 ☐a ☐b ☐c ☐d	☐0 ☐1 ☐2 ☐3 ☐4<br>☐0 ☐a ☐b ☐c ☐d	☐0 ☐1 ☐2 ☐3 ☐4<br>☐0 ☐a ☐b ☐c ☐d	☐0 ☐1 ☐2 ☐3 ☐4<br>☐0 ☐a ☐b ☐c ☐d	☐0 ☐1 ☐2 ☐3 ☐4<br>☐0 ☐a ☐b ☐c ☐d	☐0 ☐1 ☐2 ☐3 ☐4<br>☐0 ☐a ☐b ☐c ☐d
Breast	☐0 ☐1 ☐2 ☐3 ☐4<br>☐0 ☐a ☐b ☐c ☐d	☐0 ☐1 ☐2 ☐3 ☐4<br>☐0 ☐a ☐b ☐c ☐d	☐0 ☐1 ☐2 ☐3 ☐4<br>☐0 ☐a ☐b ☐c ☐d	☐0 ☐1 ☐2 ☐3 ☐4<br>☐0 ☐a ☐b ☐c ☐d	☐0 ☐1 ☐2 ☐3 ☐4<br>☐0 ☐a ☐b ☐c ☐d	☐0 ☐1 ☐2 ☐3 ☐4<br>☐0 ☐a ☐b ☐c ☐d	☐0 ☐1 ☐2 ☐3 ☐4<br>☐0 ☐a ☐b ☐c ☐d
Axilla	☐0 ☐1 ☐2 ☐3 ☐4<br>☐0 ☐a ☐b ☐c ☐d	☐0 ☐1 ☐2 ☐3 ☐4<br>☐0 ☐a ☐b ☐c ☐d	☐0 ☐1 ☐2 ☐3 ☐4<br>☐0 ☐a ☐b ☐c ☐d	☐0 ☐1 ☐2 ☐3 ☐4<br>☐0 ☐a ☐b ☐c ☐d	☐0 ☐1 ☐2 ☐3 ☐4<br>☐0 ☐a ☐b ☐c ☐d	☐0 ☐1 ☐2 ☐3 ☐4<br>☐0 ☐a ☐b ☐c ☐d	☐0 ☐1 ☐2 ☐3 ☐4<br>☐0 ☐a ☐b ☐c ☐d
Ventral midline	☐0 ☐1 ☐2 ☐3 ☐4<br>☐0 ☐a ☐b ☐c ☐d	☐0 ☐1 ☐2 ☐3 ☐4<br>☐0 ☐a ☐b ☐c ☐d	☐0 ☐1 ☐2 ☐3 ☐4<br>☐0 ☐a ☐b ☐c ☐d	☐0 ☐1 ☐2 ☐3 ☐4<br>☐0 ☐a ☐b ☐c ☐d	☐0 ☐1 ☐2 ☐3 ☐4<br>☐0 ☐a ☐b ☐c ☐d	☐0 ☐1 ☐2 ☐3 ☐4<br>☐0 ☐a ☐b ☐c ☐d	☐0 ☐1 ☐2 ☐3 ☐4<br>☐0 ☐a ☐b ☐c ☐d
Prepuce / Udder	☐0 ☐1 ☐2 ☐3 ☐4<br>☐0 ☐a ☐b ☐c ☐d	☐0 ☐1 ☐2 ☐3 ☐4<br>☐0 ☐a ☐b ☐c ☐d	☐0 ☐1 ☐2 ☐3 ☐4<br>☐0 ☐a ☐b ☐c ☐d	☐0 ☐1 ☐2 ☐3 ☐4<br>☐0 ☐a ☐b ☐c ☐d	☐0 ☐1 ☐2 ☐3 ☐4<br>☐0 ☐a ☐b ☐c ☐d	☐0 ☐1 ☐2 ☐3 ☐4<br>☐0 ☐a ☐b ☐c ☐d	☐0 ☐1 ☐2 ☐3 ☐4<br>☐0 ☐a ☐b ☐c ☐d
Limbs	☐0 ☐1 ☐2 ☐3 ☐4<br>☐0 ☐a ☐b ☐c ☐d	☐0 ☐1 ☐2 ☐3 ☐4<br>☐0 ☐a ☐b ☐c ☐d	☐0 ☐1 ☐2 ☐3 ☐4<br>☐0 ☐a ☐b ☐c ☐d	☐0 ☐1 ☐2 ☐3 ☐4<br>☐0 ☐a ☐b ☐c ☐d	☐0 ☐1 ☐2 ☐3 ☐4<br>☐0 ☐a ☐b ☐c ☐d	☐0 ☐1 ☐2 ☐3 ☐4<br>☐0 ☐a ☐b ☐c ☐d	☐0 ☐1 ☐2 ☐3 ☐4<br>☐0 ☐a ☐b ☐c ☐d
Flank	☐0 ☐1 ☐2 ☐3 ☐4<br>☐0 ☐a ☐b ☐c ☐d	☐0 ☐1 ☐2 ☐3 ☐4<br>☐0 ☐a ☐b ☐c ☐d	☐0 ☐1 ☐2 ☐3 ☐4<br>☐0 ☐a ☐b ☐c ☐d	☐0 ☐1 ☐2 ☐3 ☐4<br>☐0 ☐a ☐b ☐c ☐d	☐0 ☐1 ☐2 ☐3 ☐4<br>☐0 ☐a ☐b ☐c ☐d	☐0 ☐1 ☐2 ☐3 ☐4<br>☐0 ☐a ☐b ☐c ☐d	☐0 ☐1 ☐2 ☐3 ☐4<br>☐0 ☐a ☐b ☐c ☐d
Croup	☐0 ☐1 ☐2 ☐3 ☐4<br>☐0 ☐a ☐b ☐c ☐d	☐0 ☐1 ☐2 ☐3 ☐4<br>☐0 ☐a ☐b ☐c ☐d	☐0 ☐1 ☐2 ☐3 ☐4<br>☐0 ☐a ☐b ☐c ☐d	☐0 ☐1 ☐2 ☐3 ☐4<br>☐0 ☐a ☐b ☐c ☐d	☐0 ☐1 ☐2 ☐3 ☐4<br>☐0 ☐a ☐b ☐c ☐d	☐0 ☐1 ☐2 ☐3 ☐4<br>☐0 ☐a ☐b ☐c ☐d	☐0 ☐1 ☐2 ☐3 ☐4<br>☐0 ☐a ☐b ☐c ☐d
Tail proximal	☐0 ☐1 ☐2 ☐3 ☐4<br>☐0 ☐a ☐b ☐c ☐d	☐0 ☐1 ☐2 ☐3 ☐4<br>☐0 ☐a ☐b ☐c ☐d	☐0 ☐1 ☐2 ☐3 ☐4<br>☐0 ☐a ☐b ☐c ☐d	☐0 ☐1 ☐2 ☐3 ☐4<br>☐0 ☐a ☐b ☐c ☐d	☐0 ☐1 ☐2 ☐3 ☐4<br>☐0 ☐a ☐b ☐c ☐d	☐0 ☐1 ☐2 ☐3 ☐4<br>☐0 ☐a ☐b ☐c ☐d	☐0 ☐1 ☐2 ☐3 ☐4<br>☐0 ☐a ☐b ☐c ☐d
Tail distal	☐0 ☐1 ☐2 ☐3 ☐4<br>☐0 ☐a ☐b ☐c ☐d	☐0 ☐1 ☐2 ☐3 ☐4<br>☐0 ☐a ☐b ☐c ☐d	☐0 ☐1 ☐2 ☐3 ☐4<br>☐0 ☐a ☐b ☐c ☐d	☐0 ☐1 ☐2 ☐3 ☐4<br>☐0 ☐a ☐b ☐c ☐d	☐0 ☐1 ☐2 ☐3 ☐4<br>☐0 ☐a ☐b ☐c ☐d	☐0 ☐1 ☐2 ☐3 ☐4<br>☐0 ☐a ☐b ☐c ☐d	☐0 ☐1 ☐2 ☐3 ☐4<br>☐0 ☐a ☐b ☐c ☐d
**CALC**							
**Initials Scorer**							
