# Supplementary figures and images for: Validation of an improved insect bite hypersensitivity severity score for allergic equine insect bite hypersensitivity in horses
Source: J Vet Intern Med. 2026 Jul 6;40(4):aalag132. doi: 10.1093/jvimsj/aalag132 (PMC13336633; doi:10.1093/jvimsj/aalag132)

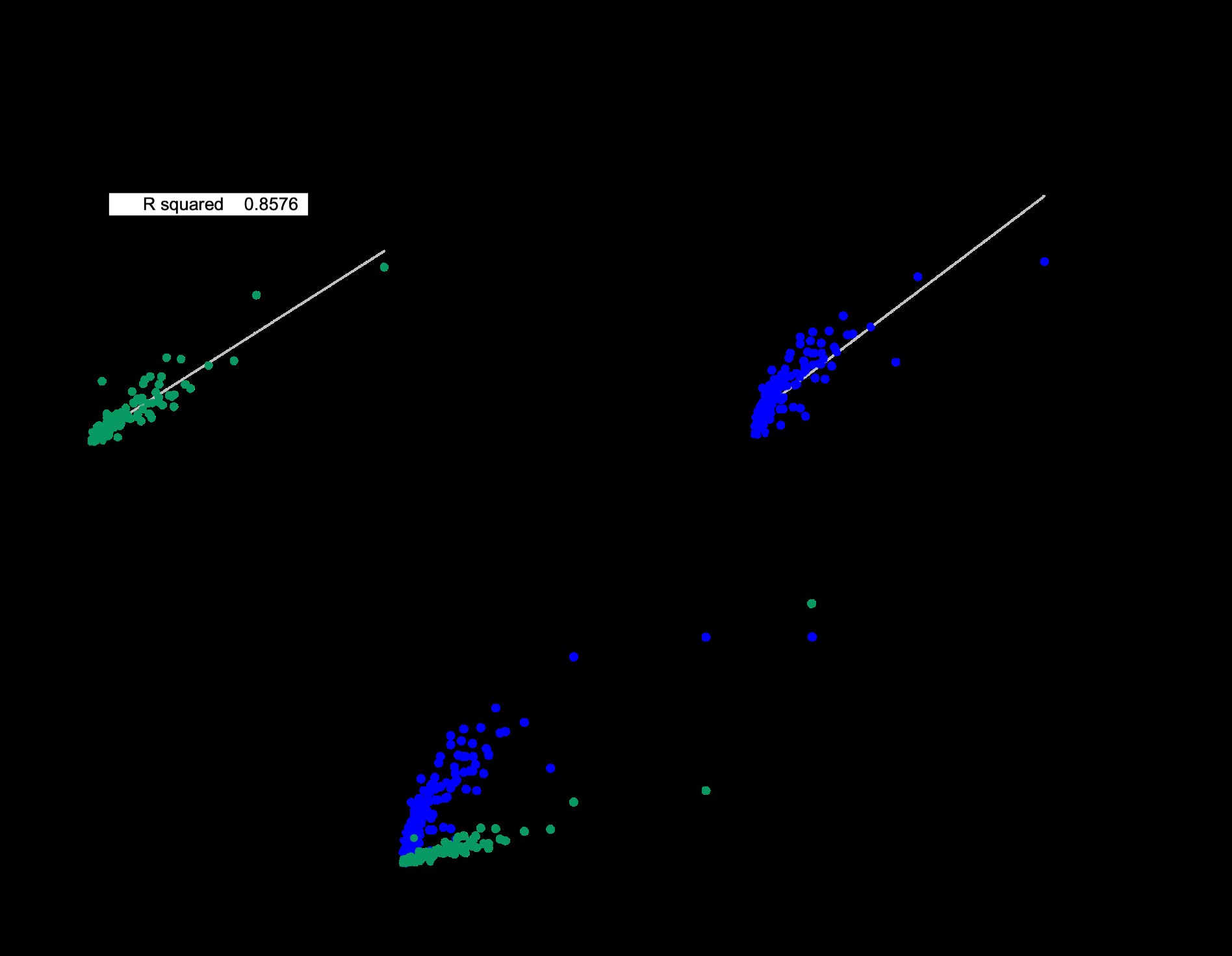

Supplement: Figure_S5_aalag132 [file figure_s5_aalag132.jpeg]
