# Supplementary material for: Validation of an improved insect bite hypersensitivity severity score for allergic equine insect bite hypersensitivity in horses
Source: J Vet Intern Med. 2026 Jul 6;40(4):aalag132. doi: 10.1093/jvimsj/aalag132 (PMC13336633; doi:10.1093/jvimsj/aalag132)
Supplement: Table_S1_aalag132 [file table_s1_aalag132.pdf]

Supplementary Table S1. ***Interobserver Pearson correlation coefficients for the lesion severity score***

Observer Pair	r	95 % CI	R squared	p-value	n
**Obs. 1 vs. Obs. 2**	0.9532	0.9055 to 0.9771	0.9087	<0.0001	32
**Obs. 1 vs. Obs. 3**	0.9528	0.8994 to 0.9782	0.9078	<0.0001	28
**Obs. 1 vs. Obs. 4**	0.9334	0.8634 to 0.9681	0.8712	<0.0001	28
**Obs. 1 vs. Obs. 5**	0.8851	0.7706 to 0.9443	0.7834	<0.0001	30
**Obs. 1 vs. Obs. 6**	0.9411	0.8788 to 0.9719	0.8857	<0.0001	30
**Obs. 2 vs. Obs. 3**	0.9133	0.8195 to 0.9595	0.8341	<0.0001	30
**Obs. 2 vs. Obs. 4**	0.9291	0.8550 to 0.9660	0.8632	<0.0001	30
**Obs. 2 vs. Obs. 5**	0.9004	0.7995 to 0.9519	0.8108	<0.0001	30
**Obs. 2 vs. Obs. 6**	0.9575	0.9117 to 0.9798	0.9168	<0.0001	30
**Obs. 3 vs. Obs. 4**	0.9282	0.8491 to 0.9665	0.8615	<0.0001	29
**Obs. 3 vs. Obs. 5**	0.8761	0.7473 to 0.9415	0.7676	<0.0001	28
**Obs. 3 vs. Obs. 6**	0.9186	0.8300 to 0.9620	0.8438	<0.0001	28
**Obs. 4 vs. Obs. 5**	0.9088	0.8155 to 0.9560	0.8258	<0.0001	30
**Obs. 4 vs. Obs. 6**	0.9487	0.8941 to 0.9756	0.9001	<0.0001	30
**Obs. 5 vs. Obs. 6**	0.9501	0.8968 to 0.9762	0.9027	<0.0001	30
