# Supplementary material for: Validation of an improved insect bite hypersensitivity severity score for allergic equine insect bite hypersensitivity in horses
Source: J Vet Intern Med. 2026 Jul 6;40(4):aalag132. doi: 10.1093/jvimsj/aalag132 (PMC13336633; doi:10.1093/jvimsj/aalag132)
Supplement: Table_S2_aalag132 [file table_s2_aalag132.pdf]

Supplementary Table S2. ***Interobserver Pearson correlation coefficients for the area score***

**Observer Pair**	**r**	**95 % CI**	**R squared**	**p-value**	**n**
**Obs. 1 vs. Obs. 2**	0.9393	0.8783 to 0.9702	0.8822	<0.0001	32
**Obs. 1 vs. Obs. 3**	0.9210	0.8347 to 0.9632	0.8483	<0.0001	28
**Obs. 1 vs. Obs. 4**	0.8966	0.7923 to 0.9500	0.8040	<0.0001	28
**Obs. 1 vs. Obs. 5**	0.8614	0.7232 to 0.9333	0.7420	<0.0001	30
**Obs. 1 vs. Obs. 6**	0.9045	0.8072 to 0.9539	0.8180	<0.0001	29
**Obs. 2 vs. Obs. 3**	0.8822	0.7589 to 0.9444	0.7782	<0.0001	30
**Obs. 2 vs. Obs. 4**	0.9010	0.8006 to 0.9522	0.8118	<0.0001	30
**Obs. 2 vs. Obs. 5**	0.8762	0.7507 to 0.9406	0.7677	<0.0001	29
**Obs. 2 vs. Obs. 6**	0.9031	0.8047 to 0.9532	0.8156	<0.0001	30
**Obs. 3 vs. Obs. 4**	0.9289	0.8505 to 0.9669	0.8628	<0.0001	28
**Obs. 3 vs. Obs. 5**	0.8634	0.7234 to 0.9352	0.7455	<0.0001	28
**Obs. 3 vs. Obs. 6**	0.8682	0.7324 to 0.9376	0.7538	<0.0001	28
**Obs. 4 vs. Obs. 5**	0.8697	0.7386 to 0.9374	0.7563	<0.0001	29
**Obs. 4 vs. Obs. 6**	0.8926	0.7846 to 0.9480	0.7967	<0.0001	30
**Obs. 5 vs. Obs. 6**	0.8849	0.7673 to 0.9450	0.7831	<0.0001	29
