# Supplementary material for: Validation of an improved insect bite hypersensitivity severity score for allergic equine insect bite hypersensitivity in horses
Source: J Vet Intern Med. 2026 Jul 6;40(4):aalag132. doi: 10.1093/jvimsj/aalag132 (PMC13336633; doi:10.1093/jvimsj/aalag132)
Supplement: Table_S3_aalag132 [file table_s3_aalag132.pdf]

**Supplementary Table S3.** ***Interobserver Pearson correlation coefficients for EqIS***

| Observer Pair | r | 95 % CI | R squared | p-value | n |
|---|---|---|---|---|---|
| **Obs. 1 vs. Obs. 2** | 0.9574 | 0.9138 to 0.9792 | 0.9166 | <0.0001 | 32 |
| **Obs. 1 vs. Obs. 3** | 0.9320 | 0.8568 to 0.9684 | 0.8686 | <0.0001 | 28 |
| **Obs. 1 vs. Obs. 4** | 0.9211 | 0.8394 to 0.9621 | 0.8484 | <0.0001 | 28 |
| **Obs. 1 vs. Obs. 5** | 0.9130 | 0.8213 to 0.9587 | 0.8335 | <0.0001 | 30 |
| **Obs. 1 vs. Obs. 6** | 0.9305 | 0.8579 to 0.9667 | 0.8659 | <0.0001 | 29 |
| **Obs. 2 vs. Obs. 3** | 0.9189 | 0.8395 to 0.9621 | 0.8443 | <0.0001 | 30 |
| **Obs. 2 vs. Obs. 4** | 0.9351 | 0.8669 to 0.9689 | 0.8744 | <0.0001 | 30 |
| **Obs. 2 vs. Obs. 5** | 0.9417 | 0.8783 to 0.9725 | 0.8867 | <0.0001 | 29 |
| **Obs. 2 vs. Obs. 6** | 0.9489 | 0.8944 to 0.9756 | 0.9004 | <0.0001 | 30 |
| **Obs. 3 vs. Obs. 4** | 0.9373 | 0.8677 to 0.9709 | 0.8786 | <0.0001 | 28 |
| **Obs. 3 vs. Obs. 5** | 0.8813 | 0.7573 to 0.9440 | 0.7768 | <0.0001 | 28 |
| **Obs. 3 vs. Obs. 6** | 0.8749 | 0.7450 to 0.9409 | 0.7654 | <0.0001 | 28 |
| **Obs. 4 vs. Obs. 5** | 0.8780 | 0.7542 to 0.9415 | 0.7709 | <0.0001 | 29 |
| **Obs. 4 vs. Obs. 6** | 0.9209 | 0.8389 to 0.9620 | 0.8480 | <0.0001 | 30 |
| **Obs. 5 vs. Obs. 6** | 0.9365 | 0.8680 to 0.9701 | 0.8771 | <0.0001 | 30 |
