# Supplementary material for: Validation of an improved insect bite hypersensitivity severity score for allergic equine insect bite hypersensitivity in horses
Source: J Vet Intern Med. 2026 Jul 6;40(4):aalag132. doi: 10.1093/jvimsj/aalag132 (PMC13336633; doi:10.1093/jvimsj/aalag132)
Supplement: Supplementary_material_aalag132 [file supplementary_material_aalag132.pdf]

Supplementary Information

Validation of an improved insect bite hypersensitivity severity score for allergic equine insect bite hypersensitivity in horses

Supplementary Methods

This section provides detailed descriptions of scoring procedures, validation protocols, and statistical methods referenced in the main Materials and Methods section.

Insect Bite Hypersensitivity (IBH) Scoring System

Lesion Severity Score (Form 24). Alopecia, blood/exudate, and crusts were classified as stand-alone lesions, whereas broken hair, scales, lichenification, and swelling were classified as non-stand-alone parameters. A lesion was defined by the presence of at least one stand-alone lesion or at least two non-stand-alone parameters within a body area. Each variable was scored according to its lesion-specific severity within the respective body area (0 = none; 1 = <25%; 2 = 25–<50%; 3 = 50–<75%; 4 = ≥75%). These percentage categories refer to the degree of involvement of each individual lesion type and do not represent the overall proportion of the body area affected, which was assessed separately using the area score (Form 35). Scores were summed per body area and across all body areas to obtain the total lesion severity score (maximum 896 points).

To ensure consistent anatomical delineation and scoring reliability, predefined region-specific rules were applied. The ventral tail base was defined as the contact between the tail base and the genital region at rest. Lateral limb surfaces were excluded from medial limb scoring. The ventral crest of the mane was delineated using anatomical landmarks including the jugular groove, the scapula, and the poll.

All scorers received standardized training in application of the lesion severity score and used a photographic reference guide to ensure scoring consistency. The original scoring form is shown in Supplementary Figure S1A–F.

Area Score (Form 35). In contrast to the lesion severity score (Form 24), the area score (Form 35) reflects the overall proportion of the body area affected by any lesion, independent of lesion

type. A body area was classified as affected according to the lesion presence rules defined for the lesion severity score (ie, at least one stand-alone lesion or two non-stand-alone variables), while the extend of all present variables accounted for the affected area, but excluding swelling and lichenification.

The proportion of affected skin within each body area was graded using a six-point ordinal scale reflecting increasing involvement: 0 = absent (0%); 1 = mild (<10%); 1.5 = mild–moderate (10–<25%); 2 = moderate (25–<33%); 2.5 = moderate–severe (33–<50%); and 3 = severe (≥50%).

Accordingly, an area score of 0 (“absent”) was assigned when no lesion presence criteria were not fulfilled, whereas “mild” involvement was assigned when at least one stand-alone lesion or two non-stand-alone variables were present and <10% of the body area was affected.

Intermediate grades (1.5 or 2.5) were recorded by marking two adjacent categories on the form. Scores were recorded per body area and used as multiplicative weighting factors for calculation of the EqIS.

Equine IBH Severity Score (EqIS; Form 24 × 35). The original scoring sheet is provided in Supplementary Figure S3A–G.

Simplified EqIS (Form 37307). The simplified EqIS scoring sheet (Form 37307) presented all predefined body areas in a condensed single-table format.

Lesion severity is graded on a 0–4 ordinal scale (0 = absent; 1 = mild; 2 = moderate; 3 = moderate–severe; 4 = severe) based on characteristic findings including broken hair, scales, alopecia, crusts, blood or exudate, swelling, or lichenification. The extent of involvement is recorded using categorical area factors (0%, <1%, 1–<25%, 25–<50%, ≥50%) corresponding to multiplicative weighting factors of 0, 0.5, 1.0, 1.25, and 1.5. For each body area, the lesion severity score is multiplied by the corresponding area factor to obtain a weighted regional score. The sum across all body areas yields the simplified EqIS.

The original scoring sheet is provided in Supplementary Figure S4.

Owner-Reported Visual Analog Scale (VAS) Scores. Owner-assessed VAS scores for overall IBH severity (I-VAS, Form 32) and pruritus intensity (P-VAS, Form 33) were recorded bi-weekly from mid March until mid October; the mean scores across the observation period were calculated for both instruments.

Each single VAS value consisted of a horizontal 10 cm line with anchors at both ends (0 = no clinical signs; 10 cm = maximum severity). Owners placed a vertical mark corresponding to the observed severity. The VAS score was defined as the distance in millimetres from the left end of the line to the mark.

Owners or caregivers familiar with the horse were permitted to perform the assessments. Standardized written instructions were provided before scoring. Completed forms were collected at scheduled visits throughout the observation period. VAS data were analyzed longitudinally and compared with clinician-assessed EqIS.

Scoring Procedures and Score Validation

Intraobserver Reliability. For intraobserver reliability assessment, both the lesion severity score and the area score were recorded at each evaluation. Intraobserver agreement was evaluated using Pearson correlation coefficients and intraclass correlation coefficients (ICC), as described in the statistical analysis section.

Interobserver Reliability. Interobserver scoring was performed under standardized field conditions during routine study visits. All scoring data were documented using standardized electronic case report forms (Prelude Dynamics, Austin, TX, USA) and archived according to good clinical practice (GCP) requirements. Interobserver agreement was analysed using Pearson correlation coefficients and ICCs, as described in the statistical analysis section.

Statistical Analysis

All statistical analyses were conducted using GraphPad Prism (version 9.0; GraphPad Software, La Jolla, CA, USA) and R (irr package). All tests were two-tailed, and significance was set at: $*p < 0.05$, $**p < 0.01$, $***p < 0.001$, $****p < 0.0001$.

Intra- and interobserver reliability for the lesion severity score and the EqIS were assessed using intraclass correlation coefficients (ICC) based on a two-way mixed-effects model with absolute agreement. The ICCs were not calculated for the area score because the scale was not intended to function as an independent composite score, but rather as a multiplicative weighting factor within the EqIS. ICC(3,1) was applied to assess intraobserver reliability and ICC(3,k) for interobserver reliability. The ICC values are reported with 95% confidence intervals.

Agreement levels were categorized as excellent (ICC ≥ 0.90), good (ICC 0.75–0.89), moderate (ICC 0.50–0.74), and poor (ICC < 0.50).

Figure Legends

Supplementary Figure S1. ***Original scoring sheet of the Lesion Severity Score (Form 24).*** (A-F) The form covers 32 predefined anatomical regions typically affected by insect bite hypersensitivity (IBH). In each region, seven lesion parameters are assessed: broken hair, self-induced alopecia, blood/exudate, scales, crusts, lichenification, and swelling/bulges. Each parameter is graded on a 0–4 ordinal scale based on defined morphological criteria and extent of involvement. Stand-alone parameters (alopecia, blood/exudate, crusts) are sufficient to define a region as lesional, whereas non-stand-alone parameters (broken hair, scales, lichenification, swelling) require at least two concurrent findings. Regional scores are summed, yielding a maximum total score of 896 per horse (32 regions × 7 parameters × 4 points).

Supplementary Figure S2. ***Original scoring sheet of the IBH Severity Assessment (Form 35).*** For the same 32 anatomical regions as in Form 24, the percentage of affected skin area is estimated and classified on a six-point ordinal scale: 0 = absent (0%), 1 = mild (<10%), 1.5 = mild–moderate (10–<25%), 2 = moderate (25–<33%), 2.5 = moderate–severe (33–<50%), 3 = severe (≥50%). If an intermediate grade (1.5 or 2.5) is intended, two adjacent boxes are ticked on the form. “Absent” is assigned when no lesions or at most one non-stand-alone parameter (broken hair, scales, lichenification, swelling) is present, whereas “mild” is given if at least one stand-alone lesion (alopecia, blood/exudate, crusts) or two non-stand-alone parameters occur. Form 35 scores are used as multiplicative weighting factors for Form 24 to generate the Equine IBH Severity Score (EqIS).

Supplementary Figure S3. *Original scoring sheet of the Combined EqIS Score (Form 24x35).* (A-G) Form 24×35 combines the lesion severity assessment from Form 24 with the regional area involvement from Form 35 for all 32 predefined body areas. The combined regional score is obtained by multiplying the Form 24 subtotal by the Form 35 area score. The sum of all weighted regional scores yields the final combined IBH score used for calculating the Equine IBH Severity Score (EqIS). Panels A–G show all pages of the original Form 24×35, including severity and area fields, multiplication fields, and the final total-score section.

Supplementary Figure S4. *Original scoring sheet of the Simplified EqIS (Form 37307).* Form 37307 represents a simplified version of the Equine IBH Severity Score (EqIS). The form integrates lesion morphology and affected area into a single streamlined table and presents the

same predefined anatomical regions as the validated EqIS in a condensed format. Lesion severity is graded on a 0–4 ordinal scale (0 = absent, 1 = mild, 2 = moderate, 3 = moderate–severe, 4 = severe) according to characteristic findings such as broken hair, scales, alopecia, blood/exudate, crusts, swelling, or lichenification. The extent of affected area is recorded using categorical factors: a = < 1% (factor x 0.5, b = 1–< 25% (factor x 1.0), c = 25–< 50% (factor x 1.25), d = ≥ 50% (factor x 1.5). The regional lesion score is multiplied by the corresponding area factor to yield a weighted regional score; the sum across all regions provides the simplified EqIS. Compared with the validated version, Form 37307 allows faster scoring and limited scorer flexibility while maintaining the modular and multiplicative logic of the EqIS system.

Supplementary Figure S5. ***Comparison of the validated, simplified, and C-Miller IBH scoring systems.*** Relationships between the validated Equine IBH Severity Score (EqIS), its simplified version, and the previously established C-Miller score were assessed using linear regression; coefficients of determination (R^2) are shown. Data were analyzed in GraphPad Prism 9.0. Each dot represents one horse (n = 142). **(A)** Validated EqIS vs. simplified EqIS (R^2 = 0.86). **(B)** Validated EqIS vs. C-Miller (R^2 = 0.73). **(C)** Direct visual comparison of all three scoring systems.
